# Wavenumber-dependent transmission of subthreshold waves on electrical synapses network model of *Caenorhabditis elegans*

**DOI:** 10.7554/eLife.99904

**Published:** 2025-01-08

**Authors:** Iksoo Chang, Taegon Chung, Sangyeol Kim

**Affiliations:** 1 Department of Brain Sciences, Daegu Gyeongbuk Institute of Science and Technology Daegu Republic of Korea; 2 Creative Research Initiative Center for Proteome Biophysics, Daegu Gyeongbuk Institute of Science and Technology Daegu Republic of Korea; 3 Supercomputing Bigdata Center, Daegu Gyeongbuk Institute of Science and Technology Daegu Republic of Korea; Georgia Institute of Technology United States; École Normale Supérieure - PSL France

**Keywords:** electrical synapses network, subthreshold oscillations, wavenumber-dependent transmission map, synchronized rhythmic activity, *C. elegans*

## Abstract

Recent experimental studies showed that electrically coupled neural networks like in mammalian inferior olive nucleus generate synchronized rhythmic activity by the subthreshold sinusoidal-like oscillations of the membrane voltage. Understanding the basic mechanism and its implication of such phenomena in the nervous system bears fundamental importance and requires preemptively the connectome information of a given nervous system. Inspired by these necessities of developing a theoretical and computational model to this end and, however, in the absence of connectome information for the inferior olive nucleus, here we investigated interference phenomena of the subthreshold oscillations in the reference system *Caenorhabditis elegans* for which the structural anatomical connectome was completely known recently. We evaluated how strongly the sinusoidal wave was transmitted between arbitrary two cells in the model network. The region of cell-pairs that are good at transmitting waves changed according to the wavenumber of the wave, for which we named a wavenumber-dependent transmission map. Also, we unraveled that (1) the transmission of all cell-pairs disappeared beyond a threshold wavenumber, (2) long distance and regular patterned transmission existed in the body-wall muscles part of the model network, and (3) major hub cell-pairs of the transmission were identified for many wavenumber conditions. A theoretical and computational model presented in this study provided fundamental insight for understanding how the multi-path constructive/destructive interference of the subthreshold oscillations propagating on electrically coupled neural networks could generate wavenumber-dependent synchronized rhythmic activity.

## Introduction

The ion current flowing through the gap junction between neurons changes the membrane potential of those neurons, which is called signal transmission by electrical synapses (Carew and Kandel, 1976). Electrical synapses have characteristics such as simple structure, fast signal transmission, bidirectional communication (in most cases), and a wide operation range for voltage including the subthreshold region (Bennett, 1997). Based on this, it has been reported that electrical synapses play various roles, such as excitation or inhibition of neurons, synchronization or desynchronization of neural activity, promotion or attenuation of rhythms, and improvement of signal-to-noise ratio (Connors, 2017). Here, rhythm refers to the periodic active pattern of neurons, and in particular, the rhythm in the subthreshold region helps neurons ignite the action potential simultaneously according to the cycle of the rhythm, and a typical example of this can be seen is the excitatory cells in inferior olivary nucleus (Bazzigaluppi et al., 2012; Benardo and Foster, 1986; Giocomo et al., 2007; Khosrovani et al., 2007; Long et al., 2002). Spontaneous and robust subthreshold membrane potential oscillations were observed in the inferior olivary cells (Benardo and Foster, 1986; Chorev et al., 2007; Devor and Yarom, 2002; Lampl and Yarom, 1997; Llinás and Yarom, 1981). The inferior olivary cells are intra-connected only by electrical synapses, and it was reported that the presence of electrical synapses is largely involved in the synchronization of subthreshold oscillations (De Zeeuw et al., 2003; Leznik and Llinás, 2005; Leznik et al., 2002; Llinás, 2013; Long et al., 2002; Turecek et al., 2016). Although electrical synapses and subthreshold oscillations are functionally closely related, theoretical and computational exploration of this has been lacking. The occurrence of subthreshold oscillations and their role in neural networks have been explored through pioneering neuron models (Roach et al., 2018; Tchumatchenko and Clopath, 2014; V-Ghaffari et al., 2016), but the establishment of an entire connectome between numerous neurons belonging to regions such as the inferior olivary nucleus remains a task to be solved in the future.

In the field of connectome, information on the indexing of all individual neurons, muscles, and organ cells present in the nematode *Caenorhabditis elegans* and the entire connectivity between those cells by chemical and electrical synapses was recently updated (Cook et al., 2019). This available connectome data of *C. elegans* identifies connectivity weights proportional to the structural size of each synapse, with 1433 electrical synapses present among a total of 469 cells, including neurons, muscles, pharynx, and other organ cells. Some examples of synchronized activity of a small number of neurons through electrical synapses in *C. elegans* have been reported. During the gentle nose touch response, nose mechanoreceptor neurons connected to electrical synapses simultaneously increased in activity, thereby serving as a coincidence detector (Chatzigeorgiou and Schafer, 2011). And a pair of GABAergic motor neurons controlling the bowel movement program repeated every 50 s used synchronized activity through electrical synapses at their axon terminal (Choi et al., 2021). However, direct observation or indirect evidence of spontaneous subthreshold oscillations has NOT been confirmed in *C. elegans*. Nevertheless, a theoretical and computational model consideration of the signal transmission of subthreshold oscillations on the entire electrical synapse network among cells with various cell types could be a great challenge.

In general, the wave properties of membrane potential are well-known, such as constructive summation of the two excitatory postsynaptic potentials or deconstructive summation of the excitatory and inhibitory postsynaptic potentials. Likewise, it is experimentally confirmed that subthreshold oscillations have wave properties as fluctuations observed in membrane potential measurements and experience interference when propagated in the neural tissue space (Chiang and Durand, 2023; Gupta et al., 2016). Therefore, interference should be considered important when subthreshold membrane potential waves propagate through the neural network and cross each other at one spatial point.

Here, we attempted to describe a situation in which wave signals, mimicking the subthreshold membrane potential waves, are propagated and interfered on a network that mimics the electrical synapse network of *C. elegans* (the cells become nodes, the electrical synapses become edges, and the subthreshold waves can flow in both directions on the edges). In the field of physics, the Anderson localization phenomenon has been well known, and it is a general wave phenomenon due to the wave interference between multiple scattering paths. Depending on the strength of the scattering of waves, the result of wave interference could be constructive or destructive, namely, waves could transmit or localize through the media, correspondingly. The theoretical and numerical method for describing the so-called Anderson localization problem usually employed the tight-binding Anderson Hamiltonian. A relevant model was developed to calculate the wave’s quantum percolation using the tight-binding Anderson Hamiltonian on a network lattice consisting of nodes and edges, where the interference by all possible propagation paths was considered when the quantum mechanical probability wave was propagated by experiencing the percolation disorder on the network lattice (Anderson, 1958; Chang et al., 1995; Meir et al., 1989; Shapir et al., 1982; Thomas and Nakanishi, 2016). In this study, we benefited from this model, and by treating the probability wave as a general wave signal and replacing the network lattice with a synapse network model we are interested in. We could calculate the interference by all possible paths which were considered when the wave signals propagate in the synapse network.

By applying the theoretical concept and computational methodology introduced above and well established in the field of physics, we calculated the signal transmission coefficient between arbitrary two cells of the model connectome as a function of the wavenumber (the quantity of inverse of wavelength) of the wave signal. Based on the signal transmission coefficients for all cell-pairs estimated under various wavenumber conditions, we unraveled (1) the existence of a threshold wavenumber above which the signal transmission disappears, (2) regions of cell-pairs with wavenumber-dependent transmission map, (3) long distance and regular patterned signal transmission, and (4) major hub cell-pair common across multiple wavenumbers. When comparing with the results for a situation that did not consider the wave interference, we revealed the role of the interference when subthreshold waves propagate on the *C. elegans*’ electrical synapse network model. Although many aspects of the actual neural network and electrical synapses were simplified or omitted in our model study, our results provided the insight of the fundamental role of the electrical synapse network with subthreshold oscillations.

## Results

### Cell-pairs in the model connectome of *C. elegans* could belong to regions where the wave signal could be transmitted (or not transmitted) below (or above) a threshold wavenumber

**Figure 1.**
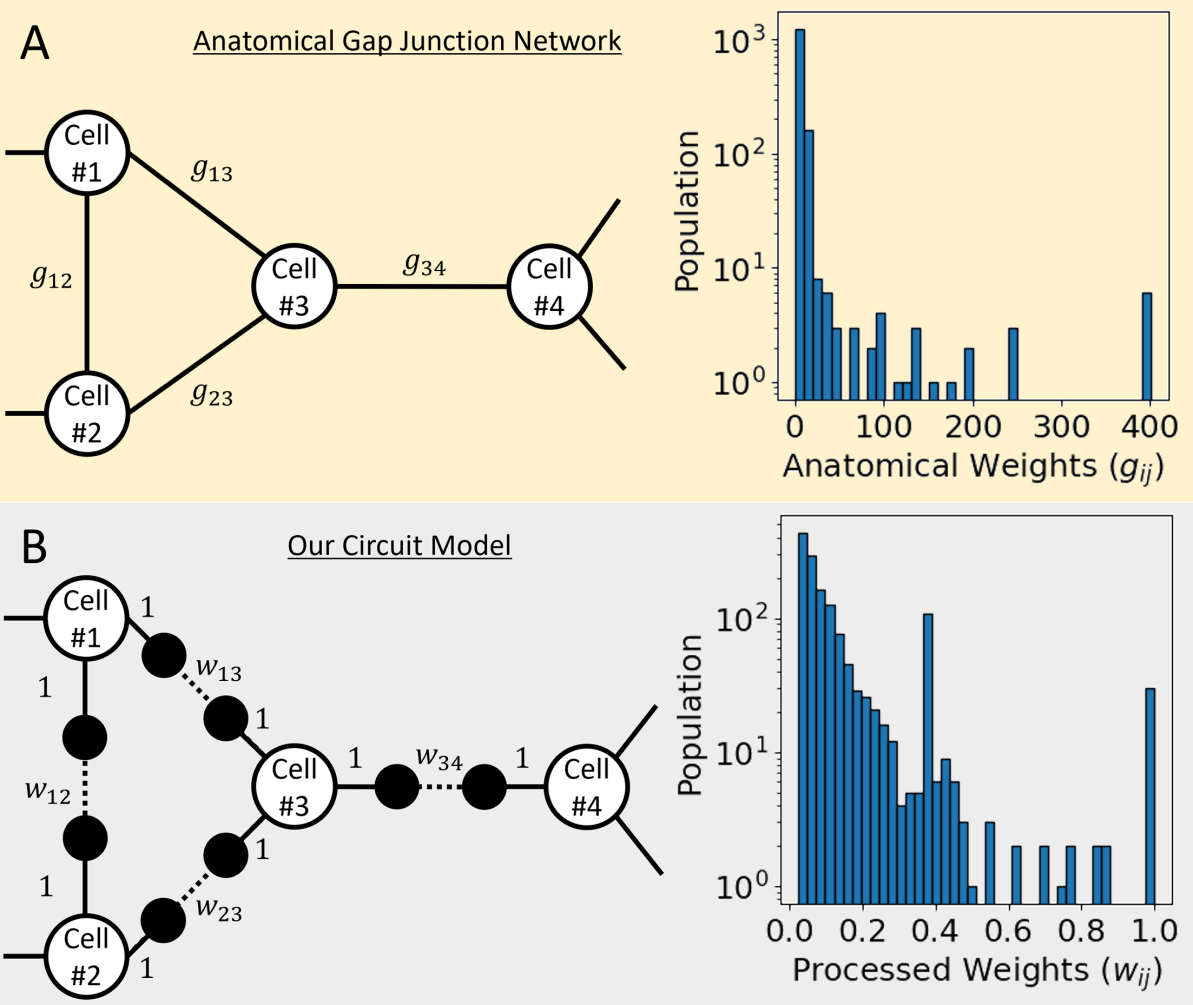
Anatomical gap junction network and our circuit model. (**A**) In the sample of the network diagram on the left, there are white-filled nodes stand for cells, and the edges of a solid line stand for the anatomical gap junction between them. The graph on the right is the distribution of 1433 gap junction weights obtained from the connectome data of *C. elegans*. (**B**) In the sample of the network diagram on the left, equally spaced two virtual nodes were added between cells connected by a gap junction, and the virtual node is presented as a black-filled circle. The solid line stands for the connection between the cell and the virtual node that gave a weight of 1, and the dotted line stands for the connection between the two virtual nodes that gave our processed weight between 0 and 1. Our processed weights are calculated from the anatomical gap junction weights, and the graph on the right shows its distribution.

In our circuit model mimicking the anatomical gap junction network of *C. elegans* (details described in ‘Construction of our circuit model’ part of Methods) (Figure 1), we calculated all the transmission coefficient $T_{ij}$ of the wave signal for each of the 109,746 cell-pairs ⟨i,j⟩ between the total 469 cells (details described in ‘Calculation for the transmission coefficient’ part of Methods). Here, the transmission coefficient was a function of energy *E* of the incoming wave, and the *E* value was a function of the wavenumber *k* of the wave signal. And according to the wavenumber, *E* value was set up to exist in the range [–10, 10], and we calculated all the $T_{ij}(E)$ of the entire cell-pairs at *E* values of 801 points listed by equal intervals over the entire range (ΔE=0.025).

**Figure 2.**
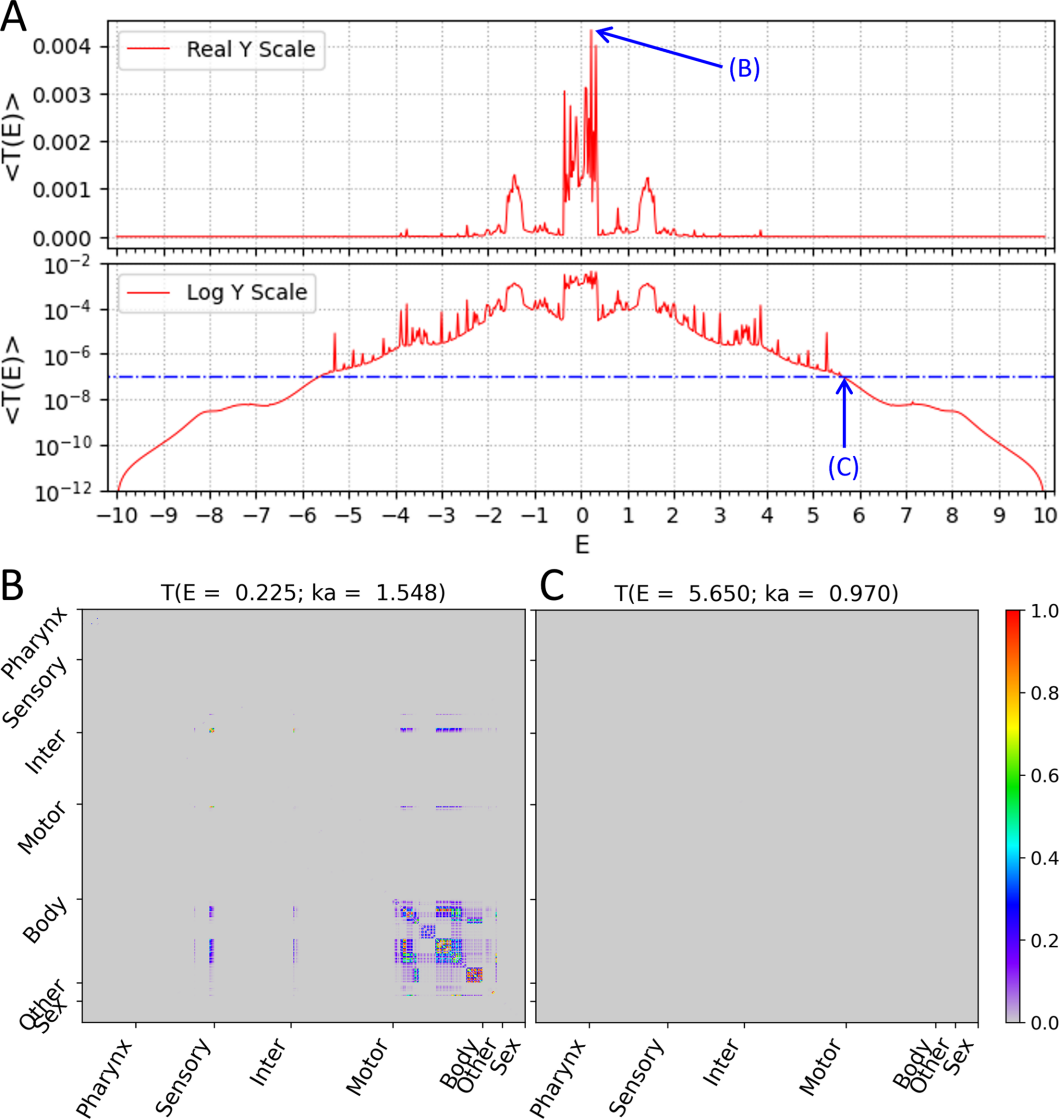
Wavenumber-dependent transmission coefficient. Average transmission coefficient for all cell-pairs as a function of *E* values (*E* value is a function of the wavenumber of the subthreshold wave). (**A**) The upper panel shows the average transmission coefficient graph on the *Y*-axis of the real scale and the lower panel shows the same graph on the *Y*-axis of the log scale. The reference value (10^–7^) of the signal mobility edge is indicated in the lower panel as a dot-dash line. (**B**) The transmission coefficient of all cell-pairs at E=0.225 is represented as a heatmap, which is the highest average transmission coefficient in our result. (**C**) The transmission coefficient of all cell-pairs at E=5.650 is represented as a heatmap, which is one of the signal mobility edges. (**B, C**) In heatmap, the *X* and *Y* axes are arranged according to the index of 469 cells, and the index follows that of the original connectome data. Instead of displaying all cell names, only seven cell types were indicated.

First, the average transmission coefficient of all cell-pairs ⟨i,j⟩ for each *E* value, $\langle T_{ij}(E)\rangle_{All\;ij\;cell-pairs}$, was obtained and drawn as a function of *E* (Figure 2A). The ⟨T(E)⟩ graph showed an incomplete but quite symmetrical form based on E=0. And on the real *Y*-axis scale, high ⟨T(E)⟩ values were broadly identified in these three ranges of the *E* values: [–1.575, –1.275], [–0.350, 0.350], and [1.275, 1.575]. Also on the log *Y*-axis scale, several relatively high ⟨T(E)⟩ values were locally identified in the form of peaks in the remaining ranges of the *E* values. Meanwhile, on the log *Y*-axis scale, the signal transmission of all cell-pairs almost disappeared in the *E* value range at both ends. In the field of solid-state physics, a threshold energy value in which electrical conductivity disappears in the energy band is called a ‘mobility edge’. We were able to define a threshold wavenumber, namely a signal mobility edge, even in the transmission of the wave signal of our circuit model. For example, considering that $\langle T(E)\rangle =10^{-7}$ is a value that can be obtained when only one cell-pair out of all cell-pairs shows 1% transmission and all others are completely unable to transmit the wave, this 10^–7^ value can be regarded as an extreme disappearance of signal transmission throughout the entire circuit. Thus, by taking this reference value, E=±5.650 in our circuit model became a signal mobility edge. If the reference value is set more conservatively lower than now, the absolute value of *E* corresponding to the signal mobility edges will be larger than now.

**Figure 2—figure supplement 1.**
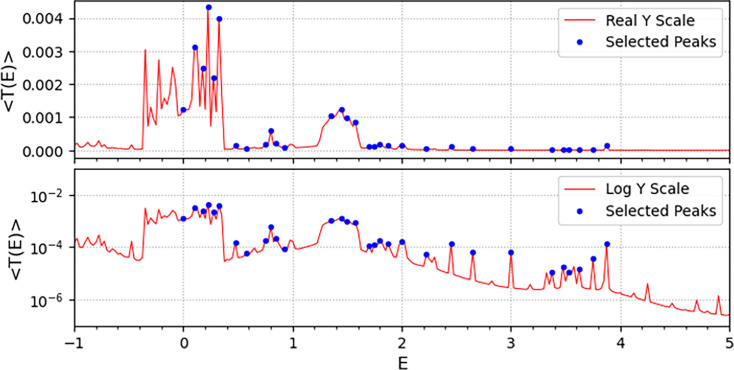
Selected ⟨T(E)⟩ peaks. The 31 selected peaks are shown as dots on the graph of the average transmission coefficient for all cell-pairs. To make the dots more visible, only the *E* value range of [–1, 5] was drawn out of the total [–10, 10]. The upper panel shows the dots and the graph on the *Y*-axis of the real scale and the lower panel shows the same things on the *Y*-axis of the log scale.

Next, the transmission coefficient of all individual cell-pairs $T_{ij}(E=0.225)$ was represented as a heatmap at the *E* value (or the corresponding wavenumber) where the ⟨T(E=0.225)⟩ value was the highest (Figure 2B). Here, we confirmed that not all cell-pairs transmit the wave signals with even transmission coefficients. Most of the strong transmissions were found in the cell-pairs of intra-body-wall muscles, and a small number of cells belonging to sensory neurons, motor neurons, and other organ cells also showed strong transmission in cell-pairs between them or to body-wall muscles. The rest of the cells except these cells were almost isolated separately without exchanging the wave signals with any cells even at this *E* value, which was the highest transmitted state on average.

In addition, the transmission coefficient of all individual cell-pairs $T_{ij}(E=5.650)$ was represented as a heatmap at the *E* value (or the corresponding wavenumber) which we defined as the signal mobility edge (Figure 2C). As expected, all cells were isolated separately without exchanging the wave signals with each other, and the entire circuit stop transmitting wave signals of this wavenumber.

### Transmission map of subthreshold waves on the electrical synapse network model depended on values of wavenumber

Between the two *E* values discussed above (E=0.225 and E=5.650), the average transmission coefficient of all cell-pairs, ⟨T(E)⟩, did not simply increase or decrease, but fluctuated along the *E* value (Figure 2). Therefore, it was expected that the composition of the cell-pairs, which showed strong transmission, also changes according to the *E* value. To efficiently investigate the changes in the composition of the cell-pairs, instead of inspecting the $T_{ij}(E)$ heatmaps in all *E* values, we selected 31 specific *E* values and tried to analyze the composition of the cell-pairs only in these *E* values. We chose the specific *E* values on the following three criteria: (1) the ⟨T(E)⟩ value was the local maximum (the shape of a peak) in the ⟨T(E)⟩ graph, (2) the components of the $T_{ij}(E)$ showed greater than 0.5 at least one cell-pair, and (3) the *E* value was positive number (Figure 2—figure supplement 1). The reasons why we used these conditions are as follows: (1) we assumed that the peak occurred in the ⟨T(E)⟩ graph due to the emergence of new cell-pairs with strong transmission, (2) we set the reference value for strong transmission to be 0.5 or higher, and (3) the *E* values where peak occurred in the ⟨T(E)⟩ graph showed left–right symmetry based on E=0. (Additional explanations are in ‘Auxiliary paragraph 1’ of Supplementary Materials.)

**Figure 3.**
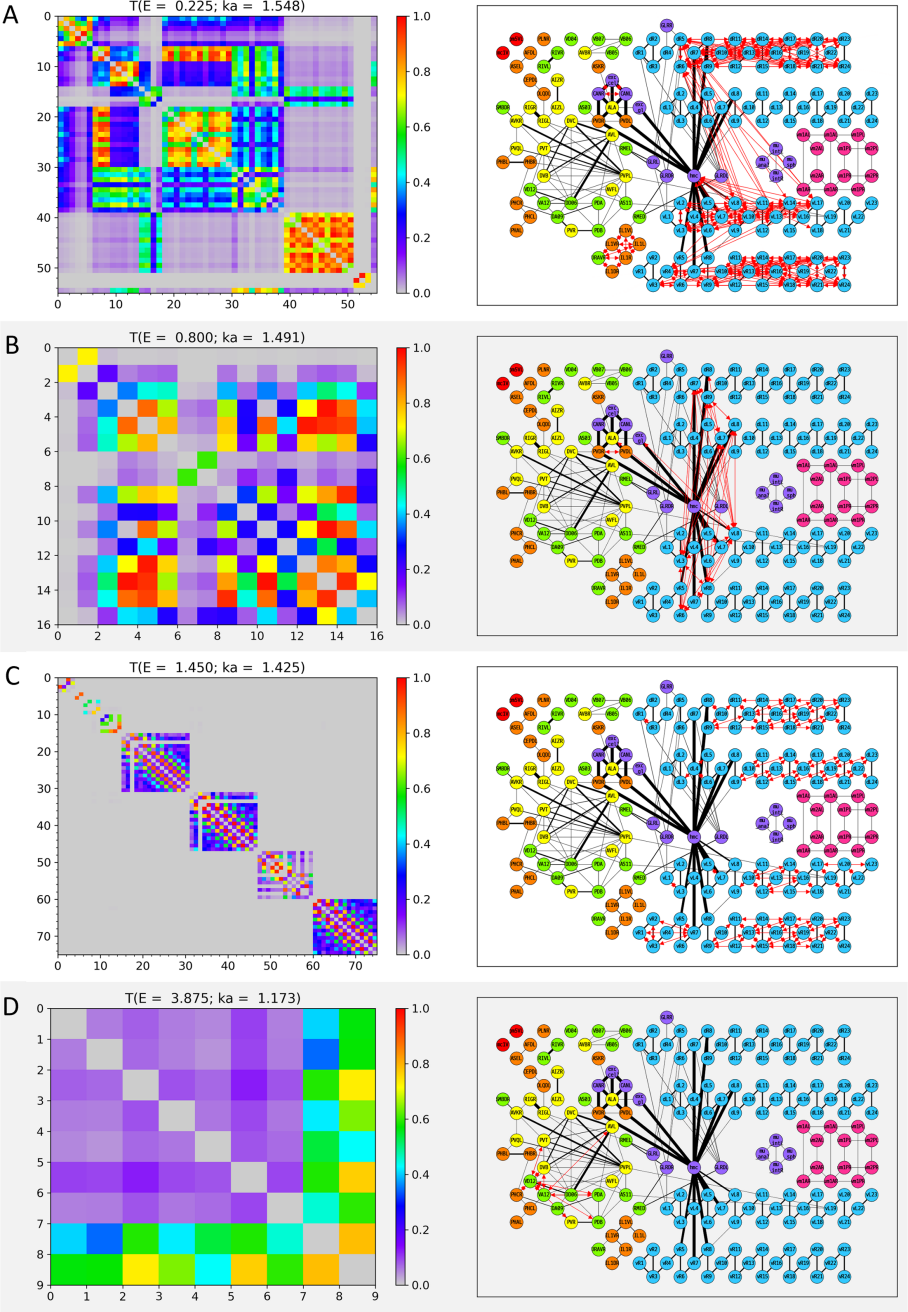
Wavenumber-dependent transmission map. The cell-pairs with strong transmission ($T_{ij}\left(E\right)>0.5$) at (**A**) E=0.225, (**B**) E=0.800, (**C**) E=1.450, and (**D**) E=3.875 (or the corresponding wavenumbers) are shown, respectively. The left panels represent the transmission coefficient between cells belonging to the cell-pairs with strong transmission at each *E* value as a heatmap. Here, the *X* and *Y* axes are the cell index, and the cell name corresponding to the cell index can be found in Appendix 1—table 1, Appendix 1—table 2, Appendix 1—table 3, Appendix 1—table 4. The right panels display the cell-pairs of strong transmission at each *E* value as red bidirectional arrows of the same thickness on the network diagram. In the network diagram, the cell name is written inside the circle that stands for each node (for the body-wall muscles, the cell name is abbreviated as follows: ex) dBWML1 → dL1, and the color of the node stands for the cell type (red: pharynx cells, orange: sensory neurons, yellow: inter neurons, green: motor neurons, light blue: body-wall muscles, purple: other end organs, magenta: sex-specific cells). Edges indicated by black solid lines stand for the electrical synapses among the cells, and the thickness of the edges is proportional to our processed weight. In the network diagram, only 173 cells that belonged to the cell-pair with strong transmission at least once in positive *E* values are represented, and the remaining cells and the electrical synapses by them are omitted from display. The virtual nodes of our circuit are also omitted from the display.

The transmission coefficients of all individual cell-pairs were represented as a heatmap at each *E* value (or the corresponding wavenumber) for the 31 specific *E* values we selected (left panels in Figure 3, Figure 3—figure supplements 1–8). To closely observe the composition of cell-pairs with strong transmission, only cells (Appendix 1—tables 1–4) belonging to the cell-pairs with $T_{ij}\left(E\right)>0.5$ were selectively exhibited in these heatmaps, not for all 469 cells. To confirm the spatial status of the cell-pairs with strong transmission on the electrical synapse network model, a network diagram was prepared first. Since drawing all 469 cells as a network diagram was complicated and poorly visible, we presented nodes for only 173 cells that belonged to the cell-pairs with $T_{ij}\left(E\right)>0.5$ in the positive *E* values. Electrical synapses between cells were represented as edges, and the thickness of the edge proportional to our processed weights ($w_{ij}$) was used. (Additional explanations are in ‘Auxiliary paragraph 2’ of Supplementary Materials). On the prepared network diagram, we marked the cell-pairs with strong transmission that satisfies $T_{ij}>0.5$ (right panels in Figure 3, Figure 3—figure supplements 1–8).

**Figure 3—figure supplement 1.**
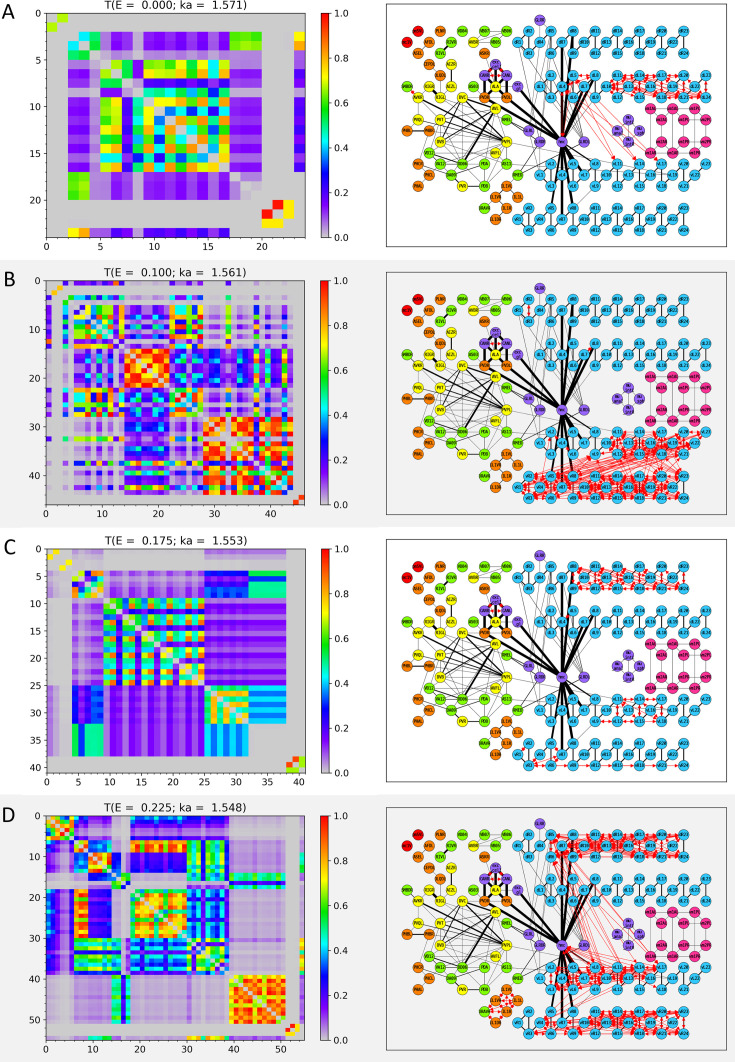
Wavenumber-dependent transmission map. The cell-pairs with strong transmission ($T_{ij}\left(E\right)>0.5$) at (**A**) E=0.000, (**B**) E=0.100, (**C**) E=0.175, and (**D**) E=0.225 (or the corresponding wavenumbers) are shown, respectively. The left panels represent the transmission coefficient between cells belonging to the cell-pairs with strong transmission at each *E* value as a heatmap. Here, the *X* and *Y* axes are the cell index, and the cell name corresponding to the cell index can be found in Appendix 1—Tables 1–4. The right panels display the cell-pairs of strong transmission at each *E* value as red bidirectional arrows of the same thickness on the network diagram. In the network diagram, the cell name is written inside the circle that stands for each node (for the body-wall muscles, the cell name is abbreviated as follows: ex) dBWML1 → dL1, and the color of the node stands for the cell type (red: pharynx cells, orange: sensory neurons, yellow: inter neurons, green: motor neurons, light blue: body-wall muscles, purple: other end organs, magenta: sex-specific cells). Edges indicated by black solid lines stand for the electrical synapses among the cells, and the thickness of the edges is proportional to our processed weight. In the network diagram, only 173 cells that belonged to the cell-pair with strong transmission at least once in positive *E* values are represented, and the remaining cells and the electrical synapses by them are omitted from display. The virtual nodes of our circuit are also omitted from the display.

**Figure 3—figure supplement 2.**
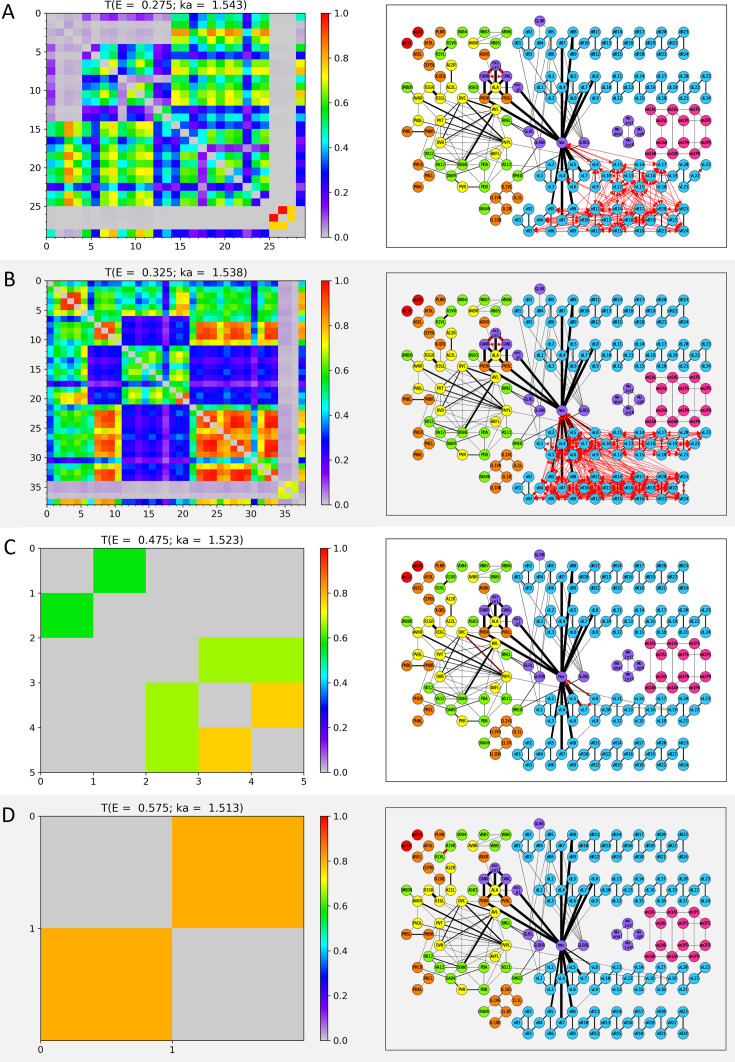
Wavenumber-dependent transmission map. The cell-pairs with strong transmission ($T_{ij}\left(E\right)>0.5$) at (**A**) E=0.275, (**B**) E=0.325, (**C**) E=0.475, and (**D**) E=0.575 (or the corresponding wavenumbers) are shown, respectively. The heatmaps of the left panels and the network diagram of the right panels are represented in the same way as in Figure 3—figure supplement 1.

**Figure 3—figure supplement 3.**
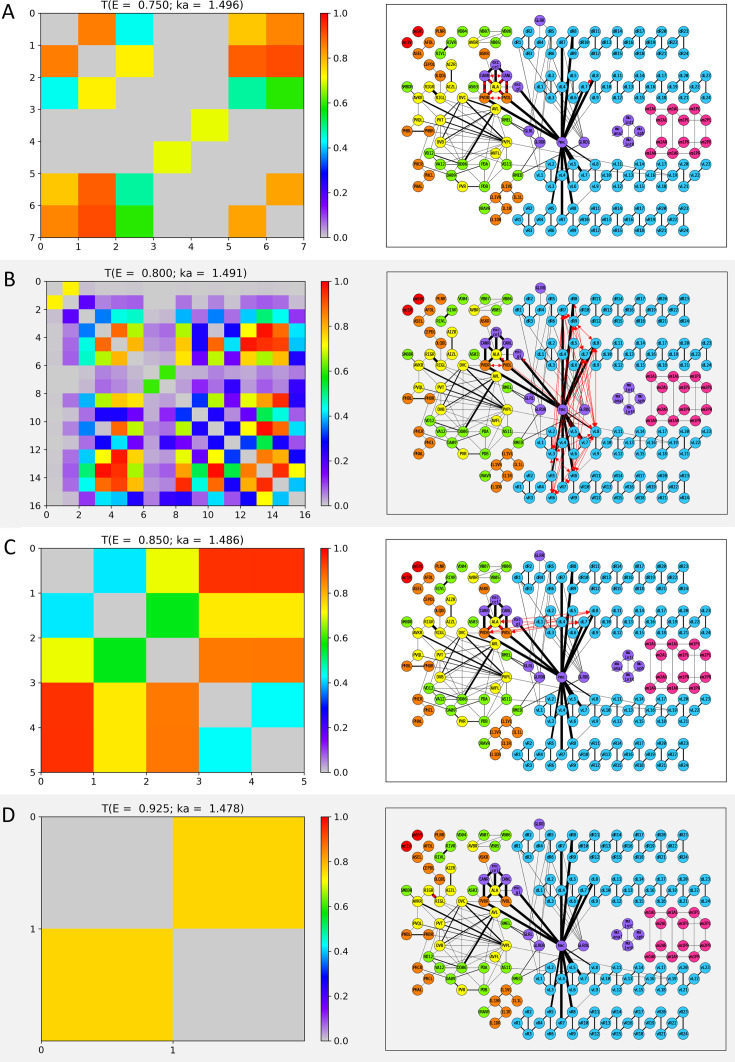
Wavenumber-dependent transmission map. The cell-pairs with strong transmission ($T_{ij}\left(E\right)>0.5$) at (**A**) E=0.750, (**B**) E=0.800, (**C**) E=0.850, and (**D**) E=0.925 (or the corresponding wavenumbers) are shown, respectively. The heatmaps of the left panels and the network diagram of the right panels are represented in the same way as in Figure 3—figure supplement 1.

**Figure 3—figure supplement 4.**
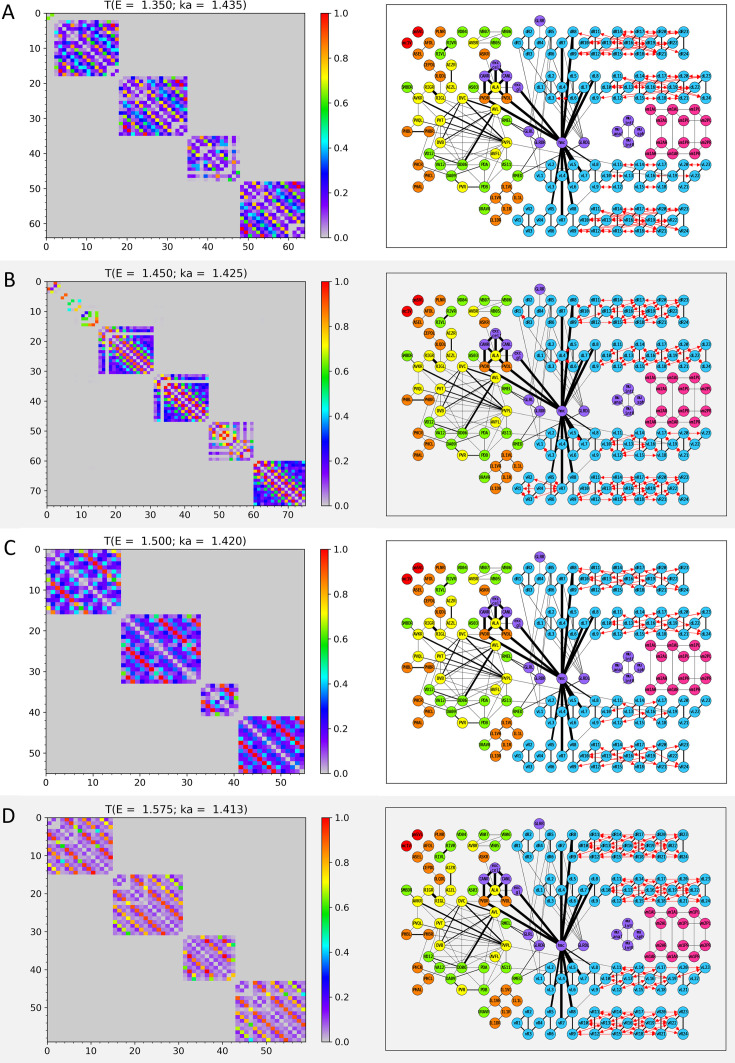
Wavenumber-dependent transmission map. The cell-pairs with strong transmission ($T_{ij}\left(E\right)>0.5$) at (**A**) E=1.350, (**B**) E=1.450, (**C**) E=1.500, and (**D**) E=1.575 (or the corresponding wavenumbers) are shown, respectively. The heatmaps of the left panels and the network diagram of the right panels are represented in the same way as in Figure 3—figure supplement 1.

**Figure 3—figure supplement 5.**
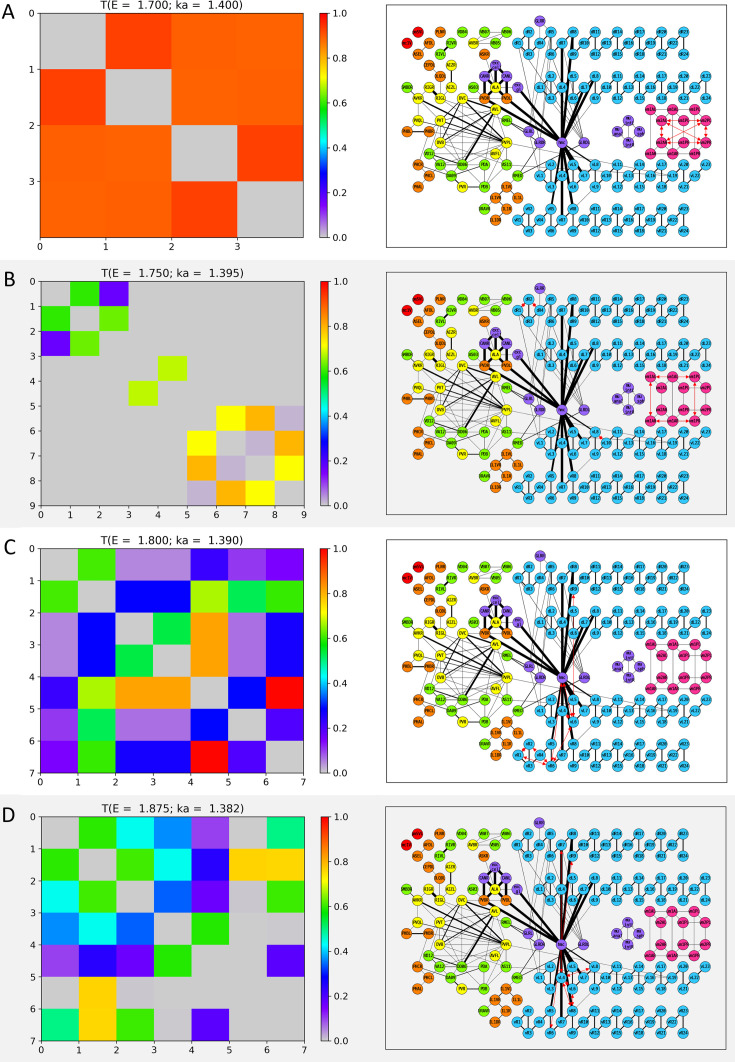
Wavenumber-dependent transmission map. The cell-pairs with strong transmission ($T_{ij}\left(E\right)>0.5$) at (**A**) E=1.700, (**B**) E=1.750, (**C**) E=1.800, and (**D**) E=1.875 (or the corresponding wavenumbers) are shown, respectively. The heatmaps of the left panels and the network diagram of the right panels are represented in the same way as in Figure 3—figure supplement 1.

**Figure 3—figure supplement 6.**
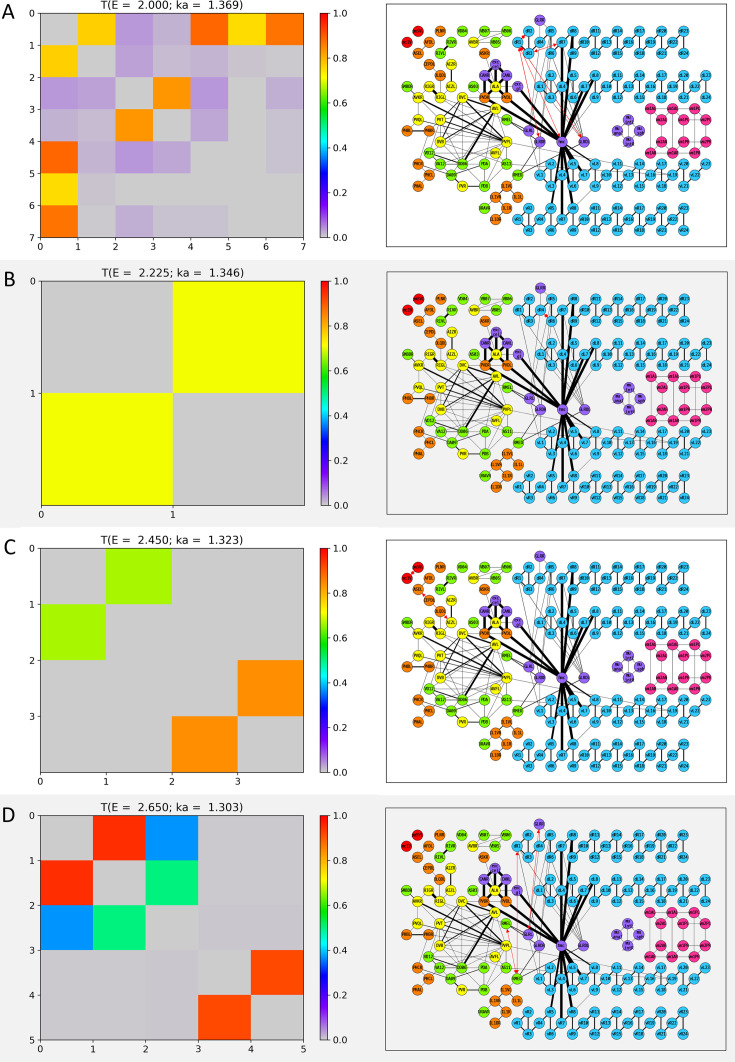
Wavenumber-dependent transmission map. The cell-pairs with strong transmission ($T_{ij}\left(E\right)>0.5$) at (**A**) E=2.000, (**B**) E=2.225, (**C**) E=2.450, and (**D**) E=2.650 (or the corresponding wavenumbers) are shown, respectively. The heatmaps of the left panels and the network diagram of the right panels are represented in the same way as in Figure 3—figure supplement 1.

**Figure 3—figure supplement 7.**
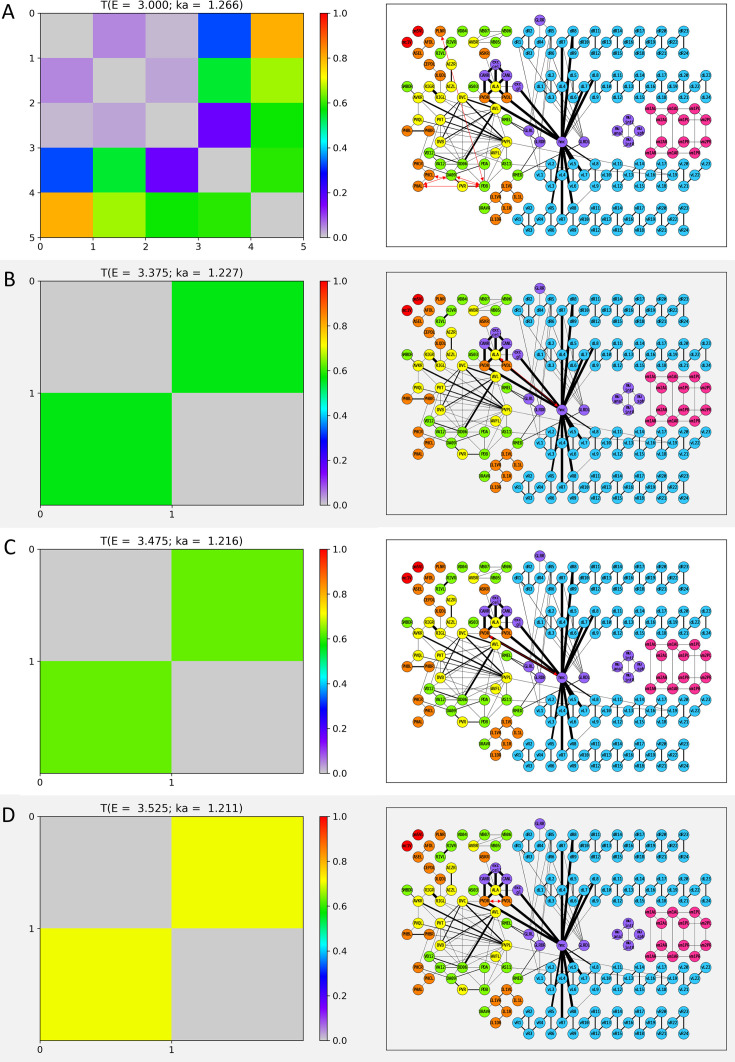
Wavenumber-dependent transmission map. The cell-pairs with strong transmission ($T_{ij}\left(E\right)>0.5$) at (**A**) E=3.000, (**B**) E=3.375, (**C**) E=3.475, and (**D**) E=3.525 (or the corresponding wavenumbers) are shown, respectively. The heatmaps of the left panels and the network diagram of the right panels are represented in the same way as in Figure 3—figure supplement 1.

**Figure 3—figure supplement 8.**
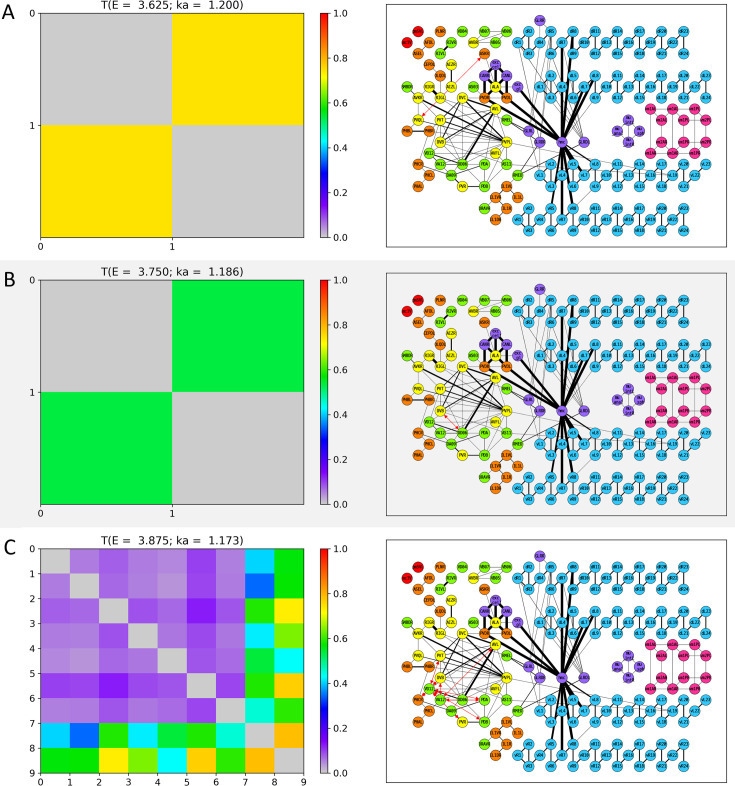
Wavenumber-dependent transmission map. The cell-pairs with strong transmission ($T_{ij}\left(E\right)>0.5$) at (**A**) E=3.625, (**B**) E=3.750, and (**C**) E=3.875 (or the corresponding wavenumbers) are shown, respectively. The heatmaps of the left panels and the network diagram of the right panels are represented in the same way as in Figure 3—figure supplement 1.

As expected above, the region of the cell-pairs with strong transmission changed depending on the value of *E* (or wavenumber), which we named ‘Wavenumber-Dependent Transmission Map (WDTM) of subthreshold waves on the electrical synapse network model’. However, it should be also noted that our WDTM was a result derived from a network where only electrical synapses were considered, not a general neural network where chemical and electrical synapses coexist.

We looked at the cell type of the cell-pairs that make up the 31 WDTMs (Figure 3, Figure 3—figure supplements 1–8). In the two *E* value ranges of [0, 0.350] and [1.275, 1.575], the high ⟨T(E)⟩ value was broadly identified (Figure 2—figure supplement 1), and many cell-pairs of intra-body-wall muscles were observed in the corresponding WDTMs of these *E* values (Figure 3A, C, Figure 3—figure supplements 1 and 2A, B, and Figure 3—figure supplement 4). Since the body-wall muscles consist of four muscle strands: dorsal/ventral-left/right strands, the cell-pair of intra-body-wall muscles can be divided into two cases: a cell-pair of intra-strand and a cell-pair of inter-strand. Following this classification, the cell-pairs of both intra- and inter-strand were found in the WDTMs of the *E* value range of [0, 0.350] (Figure 3A, Figure 3—figure supplements 1 and 2A, B), whereas only the cell-pairs of intra-strand were found in the WDTMs in the *E* value range of [1.275, 1.575] (Figure 3C and Figure 3—figure supplement 4). On the other hand, in the remaining WDTMs, a small number of cell-pairs within cells with less than 10 members were mostly found (Figure 3B, D, Figure 3—figure supplement 2C, D, Figure 3—figure supplement 3, and Figure 3—figure supplements 5–8), and the WDTMs of the two largest cases are shown in Figure 3B, D. The composition of cell-pairs with various combinations of cell types (sensory/inter/motor neurons, body-wall/sex-specific muscles, pharynx, and other organ cells) was shown in these remaining WDTMs.

### Cell-pairs in body-wall muscles on long distance with regular patterned transmission were found in the WDTMs

When looking at each WDTM on the network diagram, it shows various patterns spatially. In particular, in the case of the body-wall muscles, the cell-pairs of intra-strand existed, ranging from short distance to the longer distance than the half of the full length of the strand (Figure 3A, C). Here, the ‘Distance’ was meant by the length of the shortest path along the network between two cells of a cell-pair. If two cells are directly connected to an edge, the distance between the two cells is one. In the WDTMs of the *E* value range of [1.275, 1.575], cell-pairs with regular patterned transmission of the body-wall muscles were observed through the network diagram (Figure 3—figure supplement 4). Only the cell-pairs of intra-strand were found in these WDTMs, and the configuration of the distance of these cell-pairs was different for each WDTM (multiple of 3 at E=1.350, multiple of 2 at E=1.450, multiple of 7 at E=1.500, and multiple of 4 at E=1.575).

On the other hand, when the cell-pairs of inter-strand of the body-wall muscles appeared in the WDTMs as shown in Figure 3A, B, the head mesodermal cell (hmc) seemed to play an important role in building a bridge between them. This would be recognized from the network diagram itself of black edges without WDTM, and hmc was strongly connected near the neck of the body-wall muscles (seventh and eighth muscles) for all four strands. In the WDTM of Figure 3A, there were many cell-pairs of inter-strand between dorsal-right and ventral-left strands, and there were also many cell-pairs between the ventral-left strand and hmc, which directly revealed the role of hmc as a bridge between the two body-wall muscle strands. In the WDTM of Figure 3B, it was shown that the role of hmc was indirectly revealed as the cell-pairs of inter-strand, which existed only among the neck of four body-wall muscle strands.

Additionally, we reaffirmed the important role of hmc while confirming the distance characteristics of the cell-pairs with strong transmission. Under the conditions used when preparing the network diagram (the transmission coefficient $T_{ij}\left(E\right)$ of a cell-pair was 0.5 or higher in the range of positive *E* values at least once), a total of 146 cell-pairs with strong transmission were identified excluding the cell-pairs of intra-body-wall muscles, and their distance was investigated (Appendix 1—table 5). (Additional explanations are in ‘Auxiliary paragraph 3’ of Supplementary Materials.) Most of the cell-pairs in Appendix 1—table 5 showed the kinds of short distances 1–3, which consisted of various cell types. On the other hand, the cell-pairs with long distances in Appendix 1—table 5 ranging from 4 to 17 were mainly pairs between hmc and body-wall muscles. The strong transmission between hmc and the tail muscle, which corresponds to the longest distance, passes through the intra-strand connections from the neck to the tail of body-wall muscles.

A few cell-pairs of strong transmission with long distance in Appendix 1—table 5 turned out to be not those between hmc and the body-wall muscles. In there, one inter neuron (AVBR) and two motor neurons (AS11 and VD04) formed a cell-pair with the body-wall muscles, respectively, and four sensory neurons (CEPDL, IL1R, IL1DR, and OLQDL) formed cell-pairs between them.

### Major hub cell-pairs with the strong transmission of signals for many wavenumbers exist

**Figure 4.**
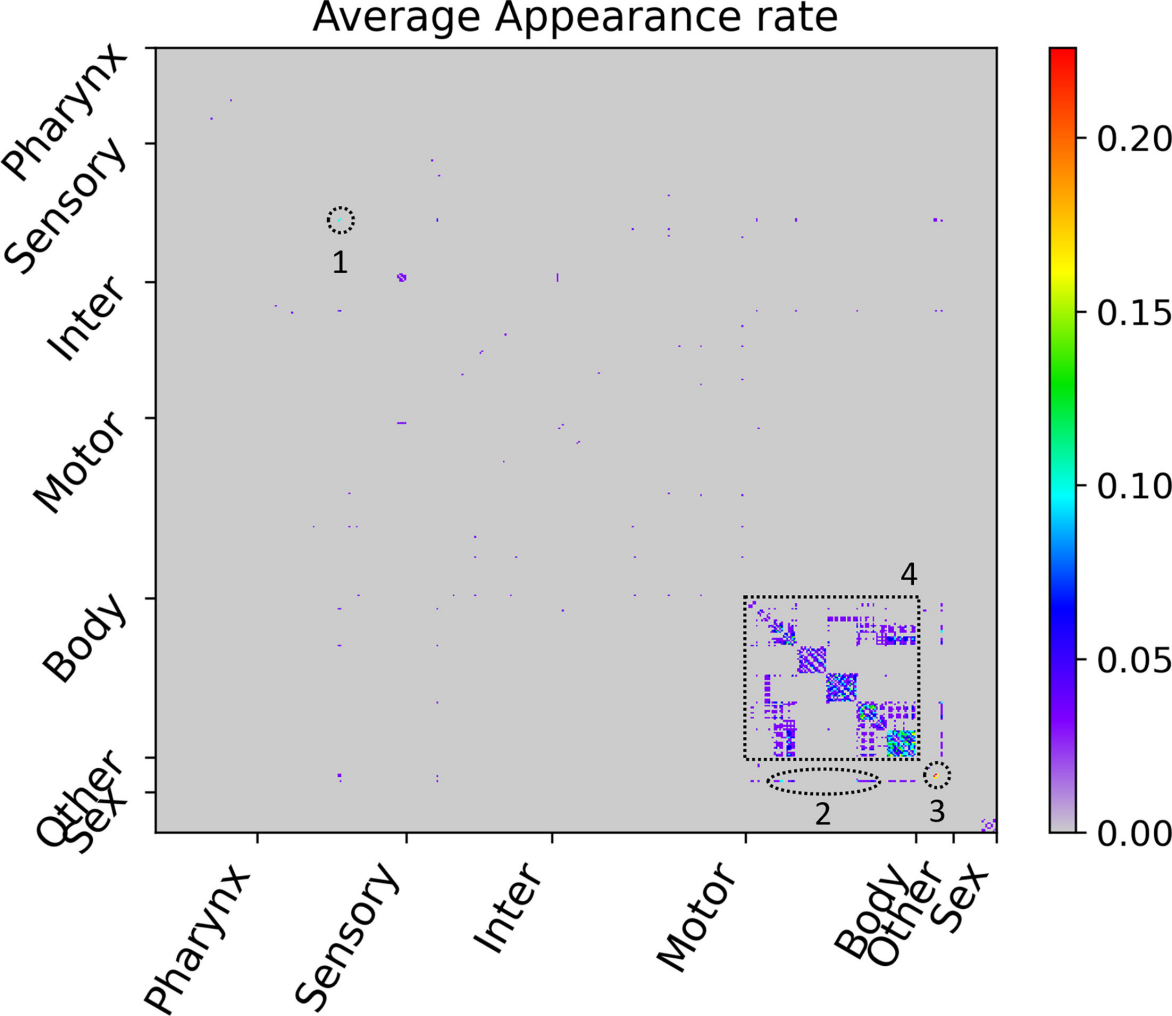
Average appearance rate as cell-pair with strong transmission in the WDTMs. The average appearance rate of all cell-pairs is represented as a heatmap. Here, the *X* and *Y* axes are arranged according to the index of 469 cells, and the index follows that of the original connectome data. Instead of displaying all cell names, only seven cell types were indicated. The highest rate is 7/31≈0.23, and four cell-pair groups showing rates of 3/31≈0.1 or higher are identified and numbered in the heatmap. (1) PVDL-PVDR pair, (2) hmc-vBWML6, hmc-vBWML7, and hmc-vBWML8 pairs, (3) CANL-CANR, CANL-exc_cell, and CANR-exc_cell pairs, and (4) many cell-pairs of intra-strand of the body-wall muscles.

We looked at cell-pairs frequently appearing in all 31 WDTMs in order to identify the major hub cell-pairs that is commonly used for various wavenumbers. First, for each cell-pair ⟨i,j⟩, we calculated the average appearance rate in all 31 WDTMs, ${\left\langle \theta \left[T_{ij}\left(E\right)-0.5\right]\right\rangle }_{E\in \left\{31\;selected\;E\right\}}$, and represented this as a heatmap (Figure 4). Where, the θ[x] was a step function, which value was 1 if 𝑥 ≥ 0 or 0 if 𝑥 < 0. Thus, the average appearance rate was 1 in the case of a cell-pair that appeared in all 31 WDTMs, and 1/31 ≈ 0.03 in the case of a cell-pair that appeared only once. In this study, we declared a cell-pair as a major hub cell-pair if the average appearance rate of a cell-pair was 𝑥 < 0 or higher. The major hub cell-pairs were the cell-pairs of intra-strand of the body-wall muscles (Figure 4). As seen in the network diagram, the edges of each strand of the body-wall muscles from the neck (seventh or eighth muscle) to the tail (twenty-third or twenty-fourth muscle) were simply connected in a line, unlike the messy edges among most neurons in the network (Figure 3). In general, the complexity of connections among most neurons increase with the number of transmissible paths of wave propagation, and the sum of phase changes coming from various path-length differences causes the deconstructive interference. Therefore, the simple (or regular) connections in each strand of the body-wall muscles from the neck to the tail reduced the number of transmissible paths of wave signals, which suppressed deconstructive interference and thus resulted in the strong transmission for various wavenumbers.

Also, the cell-pairs between hmc and the neck of the ventral-left strand of the body-wall muscles were also identified as major hub cell-pair (Figure 4). As seen in the network diagram, since the edges between hmc and the neck muscles were very strong compared to other edges (Figure 3), the relative contribution of wave signals coming from other cells could be almost ignored, and thus resulted in the strong transmission for various wavenumbers.

The cell-pairs among two sensory neurons (PVDL and PVDR) and three other organ cells (CANL, CANR, exc_cell) were also identified as major hub cell-pairs (Figure 4). The cell-pair between CANL and CANR was the most major hub cell-pair, with the highest average appearance rate of 7/31≈0.23. As seen in the network diagram, the three other organ cells were very strongly connected by the edges to each other, and the two sensory neurons were very strongly connected by the edges to both ALA inter neuron and hmc (Figure 3). Although there were cases where they were strongly connected by the edges to each other, such as between PVDL and CANL or between PVDR and CANR, they were not major hub cell-pair. Therefore, the strong edge shown on the network diagram was not a sufficient condition to implicate a major hub cell-pair.

## Discussion

The fundamental aspect of this study is that the interference phenomena of the subthreshold oscillations propagating on a neuronal circuit was described by a theoretical and computational model study of the reference system *C. elegans* for which the structural anatomical connectome was completely known. We investigated the wavenumber-dependent transmission of the sinusoidal wave signals between all cell-pairs on the connectome model of *C. elegans* by considering the interference effect caused by multi-paths of signaling.

**Figure 5.**
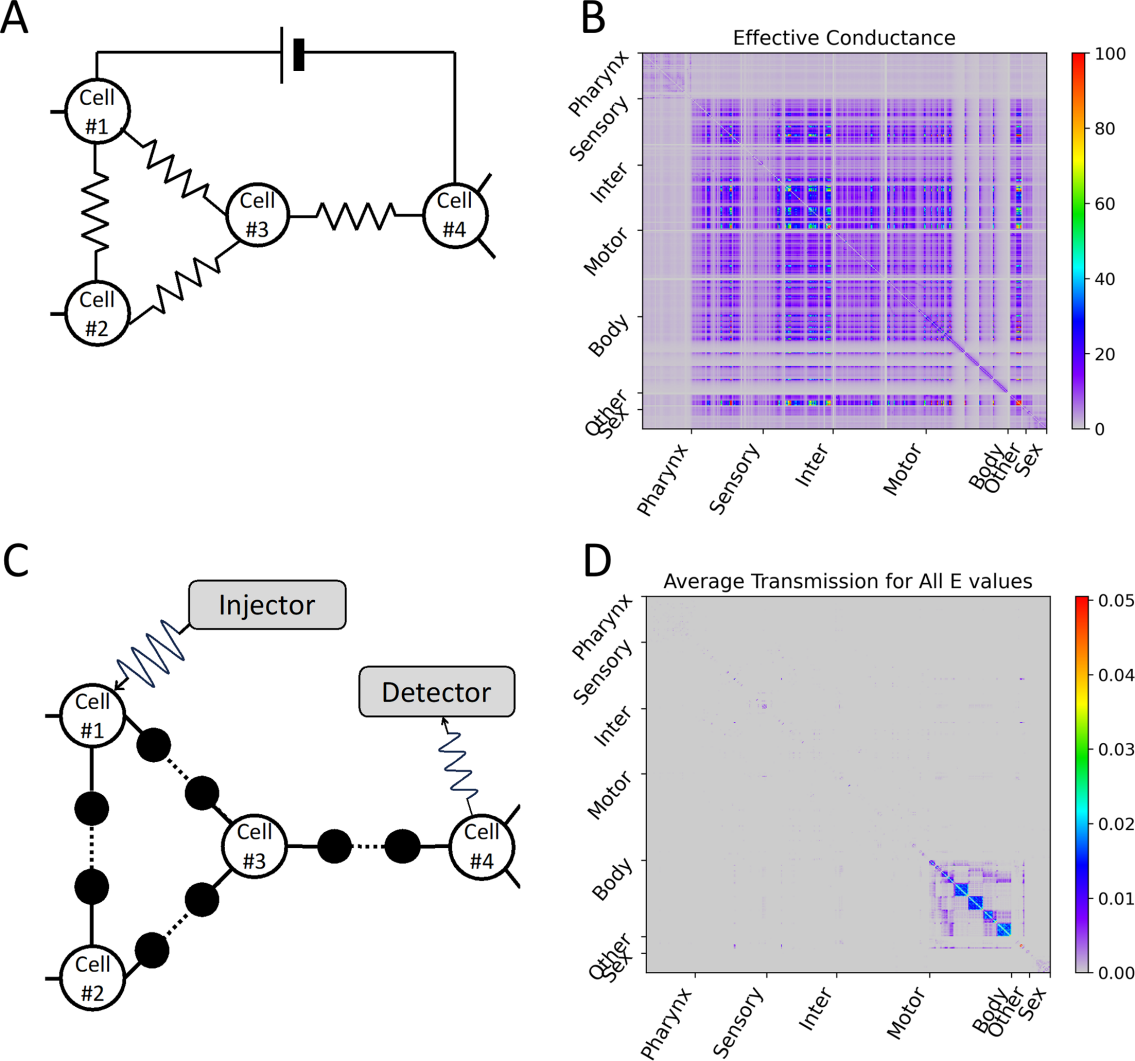
Differences depending on whether the electrical synapse network model considers interference. (**A**) In the electrical synapse network model without considering interference, when the electrical synapse is treated as an electrical resistor, the effective conductance (the reciprocal of the effective resistance) experienced by the external direct current power applied to an arbitrary cell-pair was calculated, and this value was represented as (**B**) a heatmap for all cell-pairs. (**C**) In our circuit considering interference of wave signals, the average transmission coefficient for all *E* values (or all wavenumbers) of an arbitrary cell-pair was calculated, and this value was represented as (**D**) a heatmap for all cell-pairs. (**B, D**) In heatmap, the *X* and *Y* axes are arranged according to the index of 469 cells, and the index follows that of the original connectome data. Instead of displaying all cell names, only seven cell types are indicated.

The results related to the wavenumber-dependent transmission map expanded the way we used to view signal transmission on electrical synapses. The difference in signal transmission with the presence or absence of interference effect on electrical synapses could be illustrated by considering the effective conductance between two nodes in the electric circuit with or without considering the interference effect (Figure 5). For a situation without considering the interference, we calculated the effective resistance and effective conductance for all cell-pairs by imitating a cell as a node and the electrical synapse as an electrical resistor connecting nodes as shown in Figure 5A (details in ‘Calculation for effective conductance’ part of Methods). Several inter neurons and other organ cells showed high effective conductance, while pharynx, body-wall muscles from the neck to the tail, and sex-specific muscles showed very low effective conductance (Figure 5B). Since inter neurons have many complex connections among them, many electrical current paths exist between the two inter neurons, which act as parallel connections of multiple electrical resistors. This results in lower effective resistance (or higher effective conductance). However, for a situation with considering the interference, many transmissible forward and backward paths of the signal propagation rather caused the deconstructive interference, preventing the transmission of the signal. For each cell-pair $\langle i,j\rangle$, we calculated the average transmission coefficient for all *E* values and represented this as a heatmap (Figure 5C, D). Which clearly showed that most of the neurons could not exchange wave signals with each other except for very few neurons. Wave signals were effectively delivered only within a small number of very strongly connected cell groups (PVDL/R, CANL/R, exc_cell, and hmc) or within a large number of very regularly connected cell groups (body-wall muscle strands from the neck to the tail).

In this study, we used the structural anatomical information from *C. elegans*’ electrical synapse network, but no synchronized rhythmic activity induced by subthreshold membrane potential oscillations has been reported for *C. elegans* so far. However, we aimed at investigating the signaling characteristics caused by the interference and to discover a feasible phenomenon related to the propagation of the subthreshold oscillations on a neuronal circuit of living nervous system. As to the synchronized rhythmic activation observed like in the mammalian inferior olive nucleus, we think that there might be an electrical synapse network in there that connect cells very strongly or very regularly. The plausible possibility according to our model study is that the constructive interference of subthreshold membrane potential waves with a specific wavenumber may generate the synchronized rhythmic activation. We hope that the results in our study would serve as the worthwhile framework and knowledge for designing the future experimental studies not only for inferior olive nucleus, *C. elegans* but also other living systems.

## Methods

### Construction of our circuit model

We used the contents of the ‘hermaphrodite gap jn symmetric’ tab in the ‘SI 5 Connectome adjacency matrices, corrected July 2020.xlsx’ file of the *C. elegans* connectome dataset as data for the anatomical gap junction network (Cook et al., 2019). The 469 cell names and the gap junction weight ($g_{ij}$) of all cell-pairs connected by gap junction were provided in the tab, and the weights had a value distribution from a minimum of 1 to a maximum of 401 (Figure 1A). The weights were proportional to the spatial size of the corresponding gap junction, which we hypothesized that the larger the weight, the stronger the connection between the cells and thus the better transmit the wave signal to the connected cell.

Our circuit model described *C. elegans*’ anatomical gap junction network as follows (Figure 1B). First, each cell was treated as a node in our circuit. Each gap junction connected to a cell-pair was treated as an edge connecting two nodes in our circuit, and we inserted two virtual nodes (VNs) between those two nodes to be connected sequentially through VNs (Cell-VN-VN-Cell). Here, all edges inside our circuit were set to have the identical length *a*. The edge of Cell-to-VN (or VN-to-Cell) representing like axon or dendrite of neuron gave a weight of 1, and the edge of VN-to-VN representing a gap junction gave our processed weight ($w_{ij}=\min\left\{1,\;g_{ij}/40\right\}$). Our processed weight ($w_{ij}$) was proportional to the anatomical gap junction weight ($g_{ij}$) and normalized as a value between 0 and 1 but was fixed to 1 if $g_{ij}>40$. In our circuit model, the weight of the edge meant the transmission coefficient of the signal transmitted through this edge, so if the weight was 1 corresponded to complete transmission, and 0 corresponded to complete reflection (or unavailable edge), respectively. And within our circuit, the characteristic of the signal being spontaneously attenuated, and disappearing was excluded. Therefore, when a signal incoming from the outside to a node in our circuit was reflected to the outside at the same node and simultaneously transmitted to the outside at another node, the sum of the transmission and reflection coefficients to the outside always remained at 1 without loss.

### Calculation for the transmission coefficient

We benefited from the method of calculating the transmission coefficient according to the energy *E* of the incoming wave when the electron probability wave incident on one point of the network lattice was detected on another point. This methodology, called the quantum percolation model, considered the propagation and interference of waves on the network lattice using the tight-binding Anderson Hamiltonian (Anderson, 1958; Chang et al., 1995; Meir et al., 1989; Shapir et al., 1982; Thomas and Nakanishi, 2016). In our methodology, we replaced the classical wave signals of complex number expression representing subthreshold membrane potential waves instead of the electron probability wave and replaced the network lattice with our circuit describing the electrical synapse network model built above. Then, the transmission coefficient calculated by the quantum percolation model told how well the subthreshold waves were transmitted from one cell *i* to another cell *j* while undergoing interference by multiple propagation paths in the electrical synapse network model. Here, the electrical synapses act as wave scatters that interrupt the propagation of the subthreshold waves from cell *i* to cell *j*.

In the quantum percolation model, the tight-binding Anderson Hamiltonian was defined as follows:

$$H=\sum_{m}\epsilon_{m}\left|m\right\rangle \left\langle m\right|+\sum_{\left\langle mn\right\rangle }V_{mn}\left(\left|m\right\rangle \left\langle n\right|+\left|n\right\rangle \left\langle m\right|\right),$$

where the |m⟩ (also expressed as the $\psi_{m}$) is as the electron probability wave on the *m*th node, and the ⟨m| means its complex conjugate $\psi_{m}^{*}$. We used $\psi_{m}$ as the classical wave signal observed on the *m*th node, which of complex number expression made it easy to express the phase shift of the wave and calculate the interference. The amplitude of this classical wave signal on the *m*th node was defined as the $\left\langle m|m\right\rangle =\psi_{m}^{*}\psi_{m}$. In the Hamiltonian, the $\epsilon_{m}$ is the on-site energy, and we regarded it as the resting membrane potential of the *m*th cell and set it to 0, the identical value as the relative reference value of the resting membrane potential in all cells. The $\langle mn\rangle$ represents a pair of nearest-neighbor sites in the Hamiltonian, we replaced it with a node-pair connected by the edges in our circuit. The $V_{mn}$ as a hopping matrix represents the entire structure of the network lattice in the Hamiltonian, which we implemented the structure of our circuit by setting it to $V_{mn}=1$ for a node-pair between cell and VN, and $V_{mn}=w_{mn}$ for that between two VNs. Since the total number of the cells was 469, and we inserted two VNs for every 1433 electrical synapses, the newly constructed tight-binding Anderson Hamiltonian for our circuit was represented as a matrix of 3335 by 3335.

By solving the time-independent Schrödinger equation ($H\vec{\psi }=E\vec{\psi }$) for the Hamiltonian of our circuit, we sought to obtain the reflection and transmission coefficients of the cell-pair between the IN-cell and the OUT-cell from the $\psi_{IN}$ of the IN-cell where the wave signal from the outside was incident and reflect, and the $\psi_{OUT}$ of the OUT-cell where the wave signal from our circuit transmitted to the outside. We defined $\psi_{IN}$ and $\psi_{OUT}$ as follows using complex numbers *r* and *t* with amplitudes and phases,

$$\psi_{IN}=1+r,\psi_{OUT}=t,$$

where the $\psi_{IN}$ was the sum of 1 and *r* corresponding to the incoming wave and the reflective wave, respectively, and the $\psi_{OUT}$ was *t* corresponding to the transmitted wave. The reason why the amplitude and the phase of the incoming wave on the IN-cell were 1 and 0, respectively, was because we set that as a reference amplitude and a reference phase in our circuit. The amplitude of the wave reflected from the IN-cell to the outside became the reflection coefficient with $R=r^{*}r$, and the amplitude of the wave transmitted from the OUT-cell to the outside became the transmission coefficient with $T=t^{*}t$, and T+R=1 had to be always satisfied, as mentioned above.

This methodology also assumed both an external injector and an external detector. The external injector injected the generated wave into the network lattice and received the reflective wave from the network lattice. The external detector received the transmitted wave from the network lattice. The waves on the external injector $\phi_{Injector}$ and the external detector $\phi_{Detector}$ were extended from the definitions of $\psi_{IN}$ and $\psi_{OUT}$ above, respectively, and determined as follows:

$$\phi_{Injector}=1e^{-ika}+re^{ika},\;\phi_{Detector}=te^{ika}.$$

The external injector and detector were set to be connected to the IN-cell and OUT-cell by a perfect conducting wire with a length of *a*, respectively. The wave outgoing to the external injector (detector) from the IN-cell (OUT-cell) was determined by multiplying the phase difference of $e^{ika}$ to the wave on the IN-cell (OUT-cell), whereas the wave incoming to the IN-cell from the external injector was determined by multiplying the phase difference of $e^{-ika}$ to the wave on the IN-cell. Insert these terms into the time-independent Schrödinger equation for the Hamiltonian of our circuit, the following equation was derived describing the entire system consisting of our circuit and the external injector and detector,

$$H\psi_{m}+\phi_{Injector}\delta_{m,IN}+\phi_{Detector}\delta_{m,OUT}=E\psi_{m},$$

where the *E* value on the right side representing the energy of the entire system was equivalent to the energy *E* of the incoming wave from the external injector.

In the quantum percolation model, the energy *E* of the incoming wave was defined as a tight-binding energy function as follows:

E=2γcos⁡(ka),

where γ meant the binding energy between the nearest-neighbor atoms in the tight-binding model, but we had to set it to an arbitrary value. In this study, it was set to γ=5, and the decision process was described in the below section. Applying this energy function, the above equation for the entire system was expressed as the matrices as follows:

$$\left(\begin{array}{ccc} e^{ika}-2\gamma \cos\left(ka\right) & V_{IN,m} & V_{IN,OUT}\\ V_{n,IN} & V_{nm}-2\gamma \cos\left(ka\right)\delta_{nm} & V_{n,OUT}\\ V_{OUT,IN} & V_{OUT,m} & e^{ika}-2\gamma \cos\left(ka\right) \end{array}\right)\left(\begin{array}{c} 1+r\\ \psi_{m}\\ t \end{array}\right)=\left(\begin{array}{c} e^{ika}-e^{-ika}\\ 0\\ 0 \end{array}\right),$$

where *m* and *n* meant the 3333 remaining nodes except two nodes for IN-cell and OUT-cell. For a wavenumber *k* in the range of [0,π/a] (or an *E* value in the range of [−2γ,2γ]), all components of both the left-hand square-matrix and the right-hand column-vector were fully expressed as complex numbers, and then the *r* and *t* values in the left-hand column-vector were obtained by matrix multiplication of the inverse matrix of the left-hand square-matrix and the right-hand column-vector. From squared absolute the *r* and *t* values, the reflection and transmission coefficients of the cell-pair between IN-cell and OUT-cell at the *E* value (or the corresponding wavenumber *k*) were deduced, respectively.

### Searching for optimal gamma

To determine the appropriate γ of the above energy function, we preliminarily calculated the transmission coefficient for all cell-pairs between the 469 cells under all four conditions of γ, and compared the average transmission coefficient graph as a function of the *E* value ${\left\langle T_{ij}\left(E\right)\right\rangle }_{All\;ij\;cell-pairs}$ (Appendix 1—figure 1). Where four values of 1, 2.5, 5, and 10 were attempted for γ, and equally spaced a hundred values were used for the wavenumber *k* in the range of [0,π/a]. According to the above energy function, the minimum/maximum range of the *E* value differed as [−2γ,2γ], and as γ was larger, the points of the $\langle T(E)\rangle$ graph were placed sparsely on the *X*-axis of the *E* value. In each of the $\langle T(E)\rangle$ graphs for these four conditions of γ, similarly high $\langle T(E)\rangle$ values occurred in the vicinity of E=0,±1.4. Based on this preliminarily estimation, we confirmed that the occurrence of high $\langle T(E)\rangle$ values appeared as a function of the *E* value regardless of γ. Therefore, we considered γ as just determining the minimum/maximum range of the *E* value.

The appropriate γ we wanted in this study was large enough to provide a wide *E* value range to check the signal mobility edge in the $\langle T(E)\rangle$ graph, and the appropriate γ had to be small so that the points in the $\langle T(E)\rangle$ graph did not become too sparse. It was the condition of γ=5 that satisfied this demand out of the four conditions of γ. Because the condition of γ=2.5 was not sufficient to confirm the signal mobility edge, and the condition of γ=10 had too sparse points along the *X*-axis.

### Error report on our calculation for the transmission coefficient

In principle, the sum of transmission coefficient $T_{ij}(E)$ and reflection coefficient $R_{ij}(E)$ of any cell-pair at any *E* value always had to satisfy 1 because our circuit model did not consider spontaneous attenuation of the wave signal. However, in the calculating program we wrote and used for this study, the error occurred with the sum of the two coefficients exceeding 1 (by the tolerance was 10^–5^) for a total of 5213 cell-pairs in three specific *E* values (45 cell-pairs at E=-1.000, 3836 cell-pairs at E=-0.375, and 1332 cell-pairs at E=1.000). We performed the calculations on a total of 109,746 cell-pairs for a total of 801 *E* values, so these errors correspond to 0.006% of the total calculation results. To eliminate the effect of these errors in this study, we post-corrected the two coefficients of the 5213 cell-pairs in the three specific *E* values in which the error occurred to $T_{ij}\left(E\right)=0,R_{ij}\left(E\right)=1$.

### Calculation for effective conductance

An electrical resistor network model was constructed that mimics *C. elegans*’ electrical synapse network, with the 469 cells replaced with electrical conducting nodes and the 1433 electrical synapses replaced with electrical resistors connecting these nodes. The effective resistance and its reciprocal, the effective conductance, when a direct current power source outside the electrical resistor network model contacted a cell by its positive pole, and another cell by its negative pole, respectively, were calculated (Klein and Randić, 1993). The effective conductance shows how well electric current without interference flows between the two cells contacted to the external power source.

We expressed the electric voltage at node *i* as $\nu_{i}$, the electric current flowing from node *i* to node *j* as $\mu_{ij}$, the resistance of the electrical resistor between node *i* and node *j* as $\chi_{ij}$, the conductance of that as $\omega_{ij}\left(=\chi_{ij}^{-1}\right)$. The anatomical gap junction weights ($g_{ij}$) were used as the conductance value of the electrical resistors in the network, $\omega_{ij}=g_{ij}$, with arbitrary units.

By Ohm’s law, each electrical resistor in the network satisfies the following equation,

$$\mu_{ij}=\chi_{ij}^{-1}(\nu_{i}-\nu_{j})=\omega_{ij}(\nu_{i}-\nu_{j}).$$

The sum of the electric currents entering each node by Kirchhoff’s current law satisfied the equation below,

$$\sum_{j\neq i}\mu_{ij}=\left\{ \begin{array}{l} \begin{array}{cc} \;\;\;I, & at\;i=\left(+\right) \end{array}\\ \begin{array}{cc} -I, & at\;i=\left(-\right) \end{array}\\ \begin{array}{cc} \;\;\;0, & \text{otherwise} \end{array} \end{array}\right.,$$

where (+) and (−) meant a node in which the positive and negative poles of the external power source were in contact, respectively, and the capital letter *I* was the total electric current supplied by the external power source. When Ohm’s law above was substituted on the left-hand of this equation, it was expressed as $\nu_{i}\sum_{j\neq i}\omega_{ij}-\sum_{j\neq i}\omega_{ij}\nu_{j}$, and then the equation was able to express in matrix and vector as follows:

$$L^{\omega }\vec{\nu }=I\left(\vec{e_{\left(+\right)}}-\vec{e_{\left(-\right)}}\right),$$

where $L^{\omega }$ was the Laplacian matrix of the electrical resistors’ conductance matrix (ω), defined as $L_{ij}^{\omega }=\delta_{ij}\sum_{l}\omega_{il}-\omega_{ij}$, $\vec{\nu }$ was a column-vector with an electric voltage at all nodes as a component, and $\vec{e_{\left(+\right)}}$ $\left(\vec{e_{\left(-\right)}}\right)$ was a unit column-vector of the identical dimension as $\vec{\nu }$, with only the component of the (+) node ((−) node) having a value of 1 and all other components having a value of 0. By applying the pseudo-inverse matrix of the Laplacian matrix ($L^{\omega +}$) on both sides of the equation, we obtained a particular solution as $\vec{\nu }=IL^{\omega +}\left(\vec{e_{\left(+\right)}}-\vec{e_{\left(-\right)}}\right)$.

The effective resistance applied between the positive and negative poles of the external power source was defined by Ohm’s law of the electric voltage difference between the (+) and (−) nodes and the total electric current, as follows:

$$R^{\text{eff}}=I^{-1}\left(\nu_{\left(+\right)}-\nu_{\left(-\right)}\right)=I^{-1}{\left(\vec{e_{\left(+\right)}}-\vec{e_{\left(-\right)}}\right)}^{T}\vec{\nu }.$$

Substituting here the particular solution of $\vec{\nu }$ obtained above was as follows:

$$R^{\text{eff}}={\left(\vec{e_{\left(+\right)}}-\vec{e_{\left(-\right)}}\right)}^{T}L^{\omega +}\left(\vec{e_{\left(+\right)}}-\vec{e_{\left(-\right)}}\right).$$

As a result, we were able to obtain the effective resistance for any one cell-pair by calculating the pseudo-inverse matrix of the Laplacian matrix ($L^{\omega +}$) from the electrical resistors’ conductance matrix (ω). The effective conductance was defined as the reciprocal of the effective resistance, $G^{\text{eff}}={{(R}^{\text{eff}})}^{-1}$.

## Figures and Tables

**Appendix 1—figure 1.**
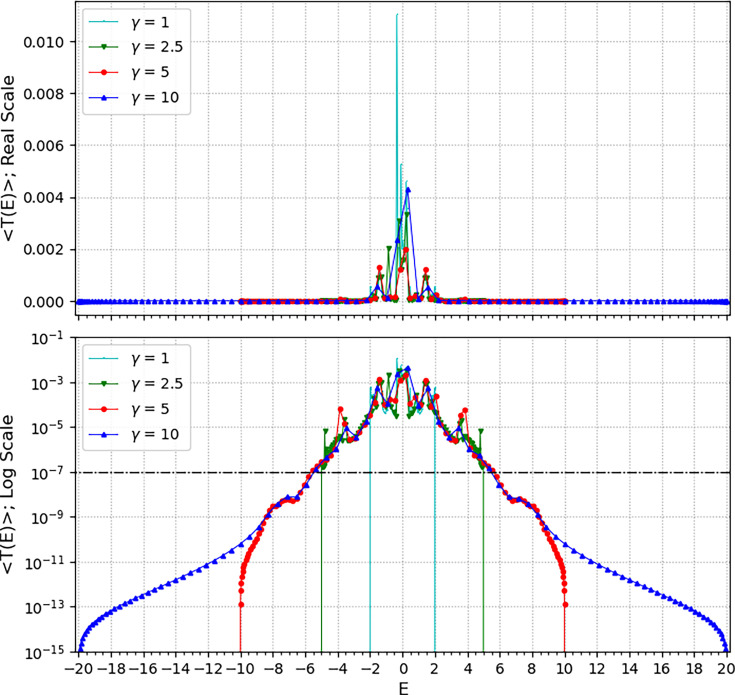
Searching for optimal gamma. The average transmission coefficient for all cell-pairs as a function of *E* value (*E* value is a function of both γ and wavenumber *k*) was calculated under the four γ conditions (1, 2.5, 5, and 10), respectively. Here, the wavenumbers used the same set as a hundred values equally spaced in the range of [0, π/a]. The upper panel shows the average transmission coefficient graphs as the *Y*-axis of the real scale and the lower panel shows the same graphs as the *Y*-axis of the log scale. The reference value (10^–7^) of the signal mobility edge is shown in the lower panel as a dot-dash line.

## Data Availability

All data generated or analyzed during this study are included in the manuscript and supporting files.

## Additional files

MDAR checklist

## Appendix 1

### Auxiliary paragraph 1

The 31 specific *E* values in this study were not fixed and were of a property that can change as a researcher adjusts the resolution of the *E* value. Perhaps the higher the resolution of the *E* value, the more specific *E* values will be found. And it was arbitrary that our reference value for strong transmission was 0.5, and this reference value can be raised or lowered depending on the researcher’s intention, and the lower the reference value, the greater the number of cell-pairs with strong transmission will be found.

### Auxiliary paragraph 2

Because of the omitted the rest of cells and their electrical synapses in the network diagram, some nodes in the diagram look like remote islands, but it should be remembered that the 173 cells were connected as one large cluster through both the drawn and the omitted electrical synapses.

### Auxiliary paragraph 3

Because the number of the cell-pairs of intra-body-wall muscles was overwhelmingly large and it was relatively easy to measure the distance compared to those of other cell-pairs, the investigation for the distance excluded them (e.g., the distance of two body-wall muscles in the same strand easily calculate from the given number in the name of the muscles).

**Appendix 1—table 1.** Cell names corresponding to the indices in the heatmaps for wavenumber-dependent transmission map. Each two columns shows the index and the corresponding cell name of the cells that belonged to the cell-pairs with strong transmission at the *E* value (or the corresponding wavenumber) indicated in the first row. The color painted on the name box stands for the cell type (red: pharynx cells, orange: sensory neurons, yellow: inter neurons, green: motor neurons, light blue: body-wall muscles, purple: other end organs, magenta: sex-specific cells).

*E* = 0.000	ka = 1.571	*E* = 0.100	ka = 1.561	*E* = 0.175	ka = 1.553	*E* = 0.225	ka = 1.548	*E* = 0.275	ka = 1.543	*E* = 0.325	ka = 1.538	*E* = 0.475	ka = 1.523	*E* = 0.575	ka = 1.513
**Index**	**Cell name**	**Index**	**Cell name**	**Index**	**Cell name**	**Index**	**Cell name**	**Index**	**Cell name**	**Index**	**Cell name**	**Index**	**Cell name**	**Index**	**Cell name**
0	AVKR	0	dBWML7	0	dBWML4	0	IL1DR	0	vBWML4	0	vBWML1	0	PVPL	0	RIVL
1	SMBDR	1	dBWMR2	1	dBWML5	1	IL1L	1	vBWMR3	1	vBWML3	1	DVC	1	RIVR
2	dBWML4	2	dBWMR3	2	vBWMR1	2	IL1R	2	vBWMR4	2	vBWML4	2	vBWML7		
3	dBWML5	3	vBWML2	3	vBWMR2	3	IL1VL	3	vBWMR5	3	vBWML5	3	vBWML8		
4	dBWML8	4	vBWML6	4	vBWMR3	4	IL1VR	4	vBWML8	4	vBWML6	4	hmc		
5	dBWML10	5	vBWMR1	5	vBWMR4	5	URAVR	5	vBWML10	5	vBWML7				
6	dBWML11	6	vBWMR2	6	vBWMR5	6	dBWMR5	6	vBWML11	6	vBWMR3				
7	dBWML12	7	vBWMR3	7	vBWMR6	7	dBWMR6	7	vBWML12	7	vBWMR4				
8	dBWML13	8	vBWMR4	8	vBWMR7	8	dBWMR7	8	vBWML13	8	vBWMR5				
9	dBWML14	9	vBWMR5	9	dBWMR8	9	vBWML2	9	vBWML15	9	vBWMR6				
10	dBWML15	10	vBWMR6	10	dBWMR9	10	vBWML3	10	vBWML16	10	vBWMR7				
11	dBWML17	11	vBWMR7	11	dBWMR10	11	vBWML4	11	vBWML17	11	vBWML10				
12	dBWML18	12	dBWML8	12	dBWMR11	12	vBWML6	12	vBWML22	12	vBWML11				
13	dBWML20	13	vBWML9	13	dBWMR12	13	vBWML7	13	vBWML23	13	vBWML12				
14	dBWML21	14	vBWML10	14	dBWMR13	14	vBWMR3	14	vBWMR10	14	vBWML16				
15	dBWML23	15	vBWML11	15	dBWMR14	15	vBWMR4	15	vBWMR11	15	vBWML17				
16	dBWML24	16	vBWML12	16	dBWMR15	16	vBWMR6	16	vBWMR12	16	vBWML18				
17	vBWML11	17	vBWML13	17	dBWMR16	17	vBWMR7	17	vBWMR14	17	vBWML19				
18	vBWML14	18	vBWML15	18	dBWMR17	18	dBWMR9	18	vBWMR15	18	vBWML21				
19	vBWML17	19	vBWML16	19	dBWMR18	19	dBWMR10	19	vBWMR16	19	vBWML22				
20	CANL	20	vBWML17	20	dBWMR19	20	dBWMR11	20	vBWMR19	20	vBWML23				
21	CANR	21	vBWML18	21	dBWMR20	21	dBWMR13	21	vBWMR20	21	vBWMR9				
22	exc_cell	22	vBWML19	22	dBWMR21	22	dBWMR14	22	vBWMR21	22	vBWMR10				
23	hmc	23	vBWML20	23	dBWMR23	23	dBWMR16	23	vBWMR23	23	vBWMR11				
		24	vBWML21	24	dBWMR24	24	dBWMR17	24	vBWMR24	24	vBWMR12				
		25	vBWML22	25	vBWML9	25	dBWMR18	25	CANL	25	vBWMR13				
		26	vBWML23	26	vBWML11	26	dBWMR20	26	CANR	26	vBWMR16				
		27	vBWMR8	27	vBWML12	27	dBWMR21	27	exc_cell	27	vBWMR17				
		28	vBWMR9	28	vBWML14	28	dBWMR23	28	hmc	28	vBWMR18				
		29	vBWMR10	29	vBWML15	29	dBWMR24			29	vBWMR19				
		30	vBWMR11	30	vBWML17	30	vBWML8			30	vBWMR20				
		31	vBWMR12	31	vBWML18	31	vBWML9			31	vBWMR22				
		32	vBWMR13	32	vBWMR9	32	vBWML10			32	vBWMR23				
		33	vBWMR14	33	vBWMR12	33	vBWML11			33	vBWMR24				
		34	vBWMR15	34	vBWMR15	34	vBWML13			34	CANL				
		35	vBWMR16	35	vBWMR18	35	vBWML14			35	CANR				
		36	vBWMR17	36	vBWMR21	36	vBWML16			36	exc_cell				
		37	vBWMR18	37	vBWMR24	37	vBWML17			37	hmc				
		38	vBWMR19	38	CANL	38	vBWML18								
		39	vBWMR20	39	CANR	39	vBWMR9								
		40	vBWMR21	40	exc_cell	40	vBWMR10								
		41	vBWMR22			41	vBWMR11								
		42	vBWMR23			42	vBWMR13								
		43	vBWMR24			43	vBWMR14								
		44	CANL			44	vBWMR16								
		45	CANR			45	vBWMR17								
						46	vBWMR18								
						47	vBWMR20								
						48	vBWMR21								
						49	vBWMR23								
						50	vBWMR24								
						51	CANL								
						52	CANR								
						53	exc_cell								
						54	hmc								

**Appendix 1—table 2.** Cell names corresponding to the indices in the heatmaps for wavenumber-dependent transmission map. It is represented in the same way as Appendix 1—table 1.

*E* = 0.750	ka = 1.496	*E* = 0.800	ka = 1.491	*E* = 0.850	ka = 1.486	*E* = 0.925	ka = 1.478	*E* = 1.350	ka = 1.435	*E* = 1.450	ka = 1.425	*E* = 1.500	ka = 1.420	*E* = 1.575	ka = 1.413
**Index**	**Cell name**	**Index**	**Cell name**	**Index**	**Cell name**	**Index**	**Cell name**	**Index**	**Cell name**	**Index**	**Cell name**	**Index**	**Cell name**	**Index**	**Cell name**
0	PVDL	0	PVDL	0	PVDL	0	RIGL	0	dBWML3	0	dBWML3	0	dBWML9	0	dBWML10
1	PVDR	1	PVDR	1	PVDR	1	RIGR	1	dBWML6	1	dBWML4	1	dBWML10	1	dBWML11
2	ALA	2	ALA	2	ALA			2	dBWML9	2	dBWML5	2	dBWML11	2	dBWML12
3	dBWML7	3	dBWML7	3	dBWML7			3	dBWML10	3	dBWML6	3	dBWML12	3	dBWML13
4	dBWML8	4	dBWMR7	4	dBWML8			4	dBWML11	4	dBWMR1	4	dBWML13	4	dBWML14
5	CANL	5	vBWML3					5	dBWML12	5	dBWMR3	5	dBWML14	5	dBWML15
6	CANR	6	vBWML5					6	dBWML13	6	vBWML1	6	dBWML15	6	dBWML16
		7	vBWML7					7	dBWML14	7	vBWML3	7	dBWML16	7	dBWML17
		8	vBWMR6					8	dBWML15	8	vBWML5	8	dBWML17	8	dBWML18
		9	vBWMR7					9	dBWML16	9	vBWML7	9	dBWML18	9	dBWML19
		10	dBWML8					10	dBWML17	10	vBWMR1	10	dBWML19	10	dBWML20
		11	dBWMR8					11	dBWML18	11	vBWMR2	11	dBWML20	11	dBWML21
		12	dBWMR9					12	dBWML19	12	vBWMR3	12	dBWML21	12	dBWML22
		13	vBWML8					13	dBWML20	13	vBWMR5	13	dBWML22	13	dBWML23
		14	vBWMR8					14	dBWML21	14	vBWMR7	14	dBWML23	14	dBWML24
		15	exc_gl					15	dBWML22	15	dBWML9	15	dBWML24	15	dBWMR9
								16	dBWML23	16	dBWML10	16	dBWMR8	16	dBWMR10
								17	dBWML24	17	dBWML11	17	dBWMR9	17	dBWMR11
								18	dBWMR8	18	dBWML12	18	dBWMR10	18	dBWMR12
								19	dBWMR9	19	dBWML13	19	dBWMR11	19	dBWMR13
								20	dBWMR10	20	dBWML14	20	dBWMR12	20	dBWMR14
								21	dBWMR11	21	dBWML15	21	dBWMR13	21	dBWMR15
								22	dBWMR12	22	dBWML16	22	dBWMR14	22	dBWMR16
								23	dBWMR13	23	dBWML17	23	dBWMR15	23	dBWMR17
								24	dBWMR14	24	dBWML18	24	dBWMR16	24	dBWMR18
								25	dBWMR15	25	dBWML19	25	dBWMR17	25	dBWMR19
								26	dBWMR16	26	dBWML20	26	dBWMR18	26	dBWMR20
								27	dBWMR17	27	dBWML21	27	dBWMR19	27	dBWMR21
								28	dBWMR18	28	dBWML22	28	dBWMR20	28	dBWMR22
								29	dBWMR19	29	dBWML23	29	dBWMR21	29	dBWMR23
								30	dBWMR20	30	dBWML24	30	dBWMR22	30	dBWMR24
								31	dBWMR21	31	dBWMR8	31	dBWMR23	31	vBWML9
								32	dBWMR22	32	dBWMR9	32	dBWMR24	32	vBWML10
								33	dBWMR23	33	dBWMR10	33	vBWML9	33	vBWML11
								34	dBWMR24	34	dBWMR11	34	vBWML10	34	vBWML12
								35	vBWML9	35	dBWMR13	35	vBWML11	35	vBWML13
								36	vBWML10	36	dBWMR14	36	vBWML12	36	vBWML14
								37	vBWML11	37	dBWMR15	37	vBWML16	37	vBWML15
								38	vBWML12	38	dBWMR16	38	vBWML17	38	vBWML16
								39	vBWML13	39	dBWMR17	39	vBWML18	39	vBWML17
								40	vBWML14	40	dBWMR18	40	vBWML19	40	vBWML18
								41	vBWML15	41	dBWMR19	41	vBWMR9	41	vBWML19
								42	vBWML16	42	dBWMR20	42	vBWMR10	42	vBWML23
								43	vBWML17	43	dBWMR21	43	vBWMR11	43	vBWMR9
								44	vBWML18	44	dBWMR22	44	vBWMR12	44	vBWMR10
								45	vBWML20	45	dBWMR23	45	vBWMR13	45	vBWMR11
								46	vBWML22	46	dBWMR24	46	vBWMR14	46	vBWMR12
								47	vBWML23	47	vBWML9	47	vBWMR16	47	vBWMR13
								48	vBWMR9	48	vBWML10	48	vBWMR17	48	vBWMR14
								49	vBWMR10	49	vBWML11	49	vBWMR18	49	vBWMR15
								50	vBWMR11	50	vBWML12	50	vBWMR19	50	vBWMR16
								51	vBWMR12	51	vBWML14	51	vBWMR20	51	vBWMR17
								52	vBWMR13	52	vBWML16	52	vBWMR21	52	vBWMR18
								53	vBWMR14	53	vBWML17	53	vBWMR23	53	vBWMR19
								54	vBWMR15	54	vBWML18	54	vBWMR24	54	vBWMR20
								55	vBWMR16	55	vBWML19			55	vBWMR21
								56	vBWMR17	56	vBWML20			56	vBWMR22
								57	vBWMR18	57	vBWML21			57	vBWMR23
								58	vBWMR19	58	vBWML22			58	vBWMR24
								59	vBWMR20	59	vBWML23				
								60	vBWMR21	60	vBWMR9				
								61	vBWMR22	61	vBWMR11				
								62	vBWMR23	62	vBWMR12				
								63	vBWMR24	63	vBWMR13				
										64	vBWMR14				
										65	vBWMR15				
										66	vBWMR16				
										67	vBWMR17				
										68	vBWMR18				
										69	vBWMR19				
										70	vBWMR20				
										71	vBWMR21				
										72	vBWMR22				
										73	vBWMR23				
										74	vBWMR24				

**Appendix 1—table 3.** Cell names corresponding to the indices in the heatmaps for wavenumber-dependent transmission map. It is represented in the same way as Appendix 1—table 1.

*E* = 1.700	ka = 1.400	*E* = 1.750	ka = 1.395	*E* = 1.800	ka = 1.390	*E* = 1.875	ka = 1.382	*E* = 2.000	ka = 1.369	*E* = 2.225	ka = 1.346	*E* = 2.450	ka = 1.323	*E* = 2.650	ka = 1.303
**Index**	**Cell name**	**Index**	**Cell name**	**Index**	**Cell name**	**Index**	**Cell name**	**Index**	**Cell name**	**Index**	**Cell name**	**Index**	**Cell name**	**Index**	**Cell name**
0	vm2AL	0	dBWMR1	0	vBWML4	0	dBWMR7	0	dBWMR1	0	dBWMR4	0	pm5VL	0	RMEL
1	vm2AR	1	dBWMR2	1	vBWML6	1	vBWML4	1	dBWMR2	1	dBWMR6	1	mc1V	1	RMED
2	vm2PL	2	dBWMR4	2	vBWMR1	2	vBWML6	2	dBWMR3			2	ASEL	2	dBWMR1
3	vm2PR	3	vBWML8	3	vBWMR2	3	vBWMR6	3	dBWMR7			3	AIZL	3	GLRL
		4	vBWML10	4	vBWMR6	4	dBWMR9	4	GLRDL					4	GLRR
		5	vm1AL	5	dBWMR9	5	vBWML8	5	GLRDR						
		6	vm1AR	6	hmc	6	vBWMR8	6	hmc						
		7	vm1PL												
		8	vm1PR												

**Appendix 1—table 4.** Cell names corresponding to the indices in the heatmaps for wavenumber-dependent transmission map. It is represented in the same way as Appendix 1—table 1.

*E* = 3.000	ka = 1.266	*E* = 3.375	ka = 1.227	*E* = 3.475	ka = 1.216	*E* = 3.525	ka = 1.211	*E* = 3.625	ka = 1.200	*E* = 3.750	ka = 1.186	*E* = 3.875	ka = 1.173
**Index**	**Cell name**	**Index**	**Cell name**	**Index**	**Cell name**	**Index**	**Cell name**	**Index**	**Cell name**	**Index**	**Cell name**	**Index**	**Cell name**
0	PLNR	0	ALA	0	PVDR	0	PVDL	0	ASKR	0	DVB	0	PHCR
1	PHAL	1	hmc	1	hmc	1	PVDR	1	PVQL	1	DD06	1	PVR
2	PHCL											2	DVB
3	DA09											3	PVT
4	PDB											4	AVL
												5	PDA
												6	PDB
												7	VA12
												8	VD12

**Appendix 1—table 5.** List of cell-pairs with strong transmission except intra body-wall muscles pairs. The first column represents the shortest distance along the network connecting the two cells of the cell-pair. The second and third columns represent the cell names of the cell-pair, and the color painted on the name box stands for the cell type (red: pharynx cells, orange: sensory neurons, yellow: inter neurons, green: motor neurons, light blue: body-wall muscles, purple: other end organs, magenta: sex-specific cells). The fourth, fifth, and sixth columns represent the transmission coefficient, the *E* value, and the wavenumber when the cell-pair shows the strongest transmission in the positive *E* value range, respectively.

Distance	Cell names		Max{*T*(*E*)}	*E*(ka)	ka	Distance	Cell names		Max{*T*(*E*)}	*E*(ka)	ka	Distance	Cell names		Max{*T*(*E*)}	*E*(ka)	ka
17	dBWMR24	hmc	0.912	0.125	1.558	3	vBWMR10	hmc	0.791	0.325	1.538	2	vm2AR	vm2PR	0.892	1.700	1.400
17	vBWMR24	hmc	0.765	0.325	1.538	3	um1AL	vm2PR	0.659	2.025	1.367	1	ASEL	AFDL	0.794	0.125	1.558
16	vBWMR23	hmc	0.797	0.325	1.538	3	um1AR	vm2PL	0.659	2.025	1.367	1	PVDL	PVDR	0.858	0.750	1.496
15	dBWMR22	hmc	0.805	0.125	1.558	3	um1PL	vm2AR	0.659	2.025	1.367	1	PVDL	ALA	0.704	0.850	1.486
15	vBWMR22	hmc	0.603	0.325	1.538	3	um1PR	vm2AL	0.659	2.025	1.367	1	PVDL	CANL	0.776	0.750	1.496
14	dBWMR21	hmc	0.946	0.125	1.558	2	pm5VL	mc1V	0.665	2.450	1.323	1	PVDR	ALA	0.728	0.750	1.496
12	dBWMR19	hmc	0.892	0.125	1.558	2	ASEL	AIZL	0.839	2.450	1.323	1	PVDR	CANR	0.914	0.750	1.496
12	vBWMR19	hmc	0.692	0.325	1.538	2	ASKR	PVQL	0.746	3.625	1.200	1	PVDR	hmc	0.627	3.475	1.216
11	dBWMR18	hmc	0.731	0.125	1.558	2	PLNR	PDB	0.802	3.000	1.266	1	PHBL	PHBR	0.852	0.375	1.533
11	vBWMR18	hmc	0.754	0.325	1.538	2	PVDL	dBWML7	0.946	0.850	1.486	1	CEPDL	OLQDL	0.639	0.050	1.566
10	dBWMR17	hmc	0.645	0.125	1.558	2	PVDL	dBWML8	0.947	0.850	1.486	1	IL1DR	IL1R	0.642	0.225	1.548
10	vBWMR17	hmc	0.789	0.325	1.538	2	PVDL	CANR	0.851	0.750	1.496	1	IL1L	IL1VL	0.599	0.225	1.548
9	dBWMR16	hmc	0.936	0.125	1.558	2	PVDR	dBWML7	0.737	0.850	1.486	1	IL1R	IL1VR	0.750	0.225	1.548
9	vBWMR16	hmc	0.739	0.325	1.538	2	PVDR	dBWML8	0.738	0.850	1.486	1	IL1VL	IL1VR	0.973	0.225	1.548
8	vBWML15	hmc	0.593	0.275	1.543	2	PVDR	CANL	0.895	0.750	1.496	1	IL1VR	URAVR	0.556	0.225	1.548
7	AVBR	dBWML12	0.601	1.000	1.471	2	PHAL	DA09	0.546	3.000	1.266	1	AIZL	AIZR	0.534	0.375	1.533
7	dBWMR14	hmc	0.867	0.125	1.558	2	PHAL	PDB	0.662	3.000	1.266	1	ALA	CANR	0.594	0.750	1.496
7	vBWML14	hmc	0.726	0.225	1.548	2	PHCL	PDB	0.577	3.000	1.266	1	PVPL	DVC	0.568	0.475	1.523
7	vBWML16	hmc	0.776	0.275	1.543	2	IL1DR	IL1L	0.781	0.225	1.548	1	DVB	DD06	0.535	3.750	1.186
6	dBWMR13	hmc	0.900	0.125	1.558	2	IL1DR	IL1VR	0.844	1.425	1.428	1	RIGL	RIGR	0.760	0.925	1.478
6	vBWML13	hmc	0.832	0.225	1.548	2	IL1L	IL1VR	0.511	0.225	1.548	1	AVKR	SMBDR	0.895	0.200	1.551
6	vBWML17	hmc	0.801	0.225	1.548	2	IL1R	IL1VL	0.824	0.225	1.548	1	PVT	VD12	0.641	3.875	1.173
6	vBWMR13	hmc	0.593	0.325	1.538	2	IL1R	URAVR	0.872	0.225	1.548	1	RIVL	RIVR	0.807	0.575	1.513
5	CEPDL	IL1R	0.716	0.050	1.566	2	IL1VL	URAVR	0.632	0.225	1.548	1	VA12	VD12	0.786	3.875	1.173
5	AS11	vBWMR4	0.574	1.000	1.471	2	ALA	hmc	0.560	3.375	1.227	1	VB05	VB06	0.950	0.050	1.566
5	VD04	vBWML5	0.617	0.075	1.563	2	PVR	VD12	0.574	3.875	1.173	1	dBWMR1	GLRDL	0.884	2.000	1.369
5	vBWML12	hmc	0.785	0.275	1.543	2	DVB	VA12	0.590	3.875	1.173	1	dBWMR1	GLRDR	0.752	2.000	1.369
5	vBWML18	hmc	0.503	0.225	1.548	2	DVB	VD12	0.734	3.875	1.173	1	vBWML5	hmc	0.790	0.325	1.538
5	vBWMR12	hmc	0.743	0.325	1.538	2	AVL	VA12	0.538	3.875	1.173	1	vBWML7	hmc	0.662	0.325	1.538
4	CEPDL	IL1DR	0.757	0.050	1.566	2	RMEL	RMED	0.949	2.650	1.303	1	vBWMR5	hmc	0.838	0.325	1.538
4	OLQDL	IL1R	0.716	0.050	1.566	2	RMED	dBWMR1	0.501	2.650	1.303	1	vBWMR7	hmc	0.573	0.325	1.538
4	VD04	vBWML7	0.535	0.075	1.563	2	DA09	PDB	0.592	3.000	1.266	1	dBWMR8	hmc	0.946	0.125	1.558
4	dBWML4	hmc	0.721	0.000	1.571	2	PDA	VA12	0.586	3.875	1.173	1	vBWML8	hmc	0.774	0.225	1.548
4	dBWMR11	hmc	0.913	0.125	1.558	2	PDA	VD12	0.766	3.875	1.173	1	CANL	exc_cell	0.762	0.250	1.546
4	vBWML11	hmc	0.705	0.275	1.543	2	PDB	VD12	0.596	3.875	1.173	1	CANR	exc_cell	0.761	0.250	1.546
4	vBWMR11	hmc	0.773	0.325	1.538	2	VB05	VB07	0.628	0.050	1.566	1	mu_intL	mu_intR	0.607	0.050	1.566
3	PHCR	VD12	0.567	3.875	1.173	2	vBWML4	hmc	0.803	0.325	1.538	1	mu_intL	mu_anal	0.984	0.050	1.566
3	OLQDL	IL1DR	0.757	0.050	1.566	2	vBWML6	hmc	0.822	0.325	1.538	1	mu_intL	mu_sph	0.984	0.050	1.566
3	IL1DR	URAVR	0.708	0.225	1.548	2	vBWMR4	hmc	0.793	0.325	1.538	1	mu_intR	mu_anal	0.625	0.050	1.566
3	IL1L	IL1R	0.859	0.225	1.548	2	vBWMR6	hmc	0.992	1.800	1.390	1	mu_intR	mu_sph	0.625	0.050	1.566
3	IL1L	URAVR	0.871	0.225	1.548	2	dBWMR9	hmc	0.807	0.125	1.558	1	um1AL	um1PL	0.573	2.025	1.367
3	ALA	dBWML7	0.866	0.850	1.486	2	vBWML8	exc_gl	0.715	0.800	1.491	1	um1AR	um1PR	0.573	2.025	1.367
3	ALA	dBWML8	0.867	0.850	1.486	2	vBWML9	hmc	0.707	0.225	1.548	1	vm2AL	vm2AR	0.927	1.700	1.400
3	ALA	vBWML8	0.507	0.800	1.491	2	GLRL	GLRR	0.916	2.650	1.303	1	vm1AL	vm1AR	0.714	1.750	1.395
3	AVFL	AS03	0.625	1.000	1.471	2	CANL	CANR	0.999	1.950	1.375	1	vm1AL	vm1PL	0.795	1.750	1.395
3	dBWML5	hmc	0.806	0.000	1.571	2	mu_anal	mu_sph	0.984	0.050	1.566	1	vm1AR	vm1PR	0.795	1.750	1.395
3	dBWMR1	hmc	0.869	2.000	1.369	2	vm2AL	vm2PL	0.892	1.700	1.400	1	vm1PL	vm1PR	0.714	1.750	1.395
3	vBWML3	hmc	0.604	0.325	1.538	2	vm2AL	vm2PR	0.888	1.700	1.400	1	vm2PL	vm2PR	0.927	1.700	1.400
3	vBWML10	hmc	0.797	0.225	1.548	2	vm2AR	vm2PL	0.888	1.700	1.400						
